# Circulating hsa-miR-5096 predicts ^18^F-FDG PET/CT positivity and modulates somatostatin receptor 2 expression: a novel miR-based assay for pancreatic neuroendocrine tumors

**DOI:** 10.3389/fonc.2023.1136331

**Published:** 2023-05-23

**Authors:** Martine Bocchini, Marcella Tazzari, Sara Ravaioli, Filippo Piccinini, Flavia Foca, Michela Tebaldi, Fabio Nicolini, Ilaria Grassi, Stefano Severi, Raffaele Adolfo Calogero, Maddalena Arigoni, Joerg Schrader, Massimiliano Mazza, Giovanni Paganelli

**Affiliations:** ^1^ Immunotherapy, Cell Therapy and Biobank (ITCB), IRCCS Istituto Romagnolo per lo Studio dei Tumori (IRST) “Dino Amadori”, Meldola, Italy; ^2^ Biosciences Laboratory, IRCCS Istituto Romagnolo per lo Studio dei Tumori (IRST) “Dino Amadori”, Meldola, Italy; ^3^ Scientific Directorate, IRCCS Istituto Romagnolo per lo Studio dei Tumori (IRST) “Dino Amadori”, Meldola, Italy; ^4^ Department of Medical and Surgical Sciences (DIMEC), University of Bologna, Bologna, Italy; ^5^ Unit of Biostatistics and Clinical Trials, IRCCS Istituto Romagnolo per lo Studio dei Tumori (IRST) “Dino Amadori”, Meldola, Italy; ^6^ Nuclear Medicine and Radiometabolic Unit, IRCCS Istituto Romagnolo per lo Studio dei Tumori (IRST) “Dino Amadori”, Meldola, Italy; ^7^ Molecular Biotechnology Center, Department of Biotechnology and Health Sciences, University of Turin, Turin, Italy; ^8^ Department of Medicine, University Medical Center Hamburg-Eppendorf, Hamburg, Germany

**Keywords:** pancreatic neuroendocrine tumors, PRRT (peptide receptor radionuclide therapy), miRNA – microRNA, functional imaging (positron-emission tomography), SSTR2

## Abstract

Gastro-entero-pancreatic neuroendocrine tumors (GEP-NETs) are rare diseases encompassing pancreatic (PanNETs) and ileal NETs (SINETs), characterized by heterogeneous somatostatin receptors (SSTRs) expression. Treatments for inoperable GEP-NETs are limited, and SSTR-targeted Peptide Receptor Radionuclide Therapy (PRRT) achieves variable responses. Prognostic biomarkers for the management of GEP-NET patients are required. ^18^F-FDG uptake is a prognostic indicator of aggressiveness in GEP-NETs. This study aims to identify circulating and measurable prognostic miRNAs associated with ^18^F-FDG-PET/CT status, higher risk and lower response to PRRT.

**Methods:**

Whole miRNOme NGS profiling was conducted on plasma samples obtained from well-differentiated advanced, metastatic, inoperable G1, G2 and G3 GEP-NET patients enrolled in the non-randomized LUX (NCT02736500) and LUNET (NCT02489604) clinical trials prior to PRRT (screening set, n= 24). Differential expression analysis was performed between ^18^F-FDG positive (n=12) and negative (n=12) patients. Validation was conducted by Real Time quantitative PCR in two distinct well-differentiated GEP-NET validation cohorts, considering the primary site of origin (PanNETs n=38 and SINETs n=30). The Cox regression was applied to assess independent clinical parameters and imaging for progression-free survival (PFS) in PanNETs. *In situ* RNA hybridization combined with immunohistochemistry was performed to simultaneously detect miR and protein expression in the same tissue specimens. This novel semi-automated miR-protein protocol was applied in PanNET FFPE specimens (n=9). *In vitro* functional experiments were performed in PanNET models.

**Results:**

While no miRNAs emerged to be deregulated in SINETs, hsa-miR-5096, hsa-let-7i-3p and hsa-miR-4311 were found to correlate with ^18^F-FDG-PET/CT in PanNETs (p-value:<0.005). Statistical analysis has shown that, hsa-miR-5096 can predict 6-month PFS (p-value:<0.001) and 12-month Overall Survival upon PRRT treatment (p-value:<0.05), as well as identify ^18^F-FDG-PET/CT positive PanNETs with worse prognosis after PRRT (p-value:<0.005). In addition, hsa-miR-5096 inversely correlated with both SSTR2 expression in PanNET tissue and with the ^68^Gallium-DOTATOC captation values (p-value:<0.05), and accordingly it was able to decrease *SSTR2* when ectopically expressed in PanNET cells (p-value:<0.01).

**Conclusions:**

hsa-miR-5096 well performs as a biomarker for ^18^F-FDG-PET/CT and as independent predictor of PFS. Moreover, exosome-mediated delivery of hsa-miR-5096 may promote SSTR2 heterogeneity and thus resistance to PRRT.

## Introduction

Gastro-entero-pancreatic neuroendocrine tumors (GEP-NETs) are rare and heterogeneous malignancies arising from the neuroendocrine system, encompassing pancreatic (PanNETs) and ileal NETs (SINETs). NET disease exhibits variable aggressiveness depending on the site of origin, grade, stage, and functionality (1). PanNETs represent less than 5% of all pancreatic cancers, although incidence and prevalence are rising (2). PanNETs include tumors with a wide spectrum of clinical behaviors, often indolent and diagnosed in advanced stage (3). Nowadays, PanNETs can be diagnosed earlier and updated therapeutic algorithms and guidelines have been proposed (4–10). Latest updates in the 5th edition (2019) of the World Health Organization (WHO) identify a novel G3 PanNET molecular subset, which includes well-differentiated tumors with high proliferative index (>20%) (11). Despite novel classification helping the stratification of patients, improving prognosis and response to treatment (12, 13), substantial differences in clinical behavior and biology still remain, making personalized treatment and prognostication challenging for advanced PanNETs (6, 14, 15). Somatostatin receptors (SSTRs) are expressed by 80% of GEP-NETs, and PanNETs display heterogeneous patterns of SSTRs expression from 50% to 100%, with isoform 2 being the most prevalent one (16). Peptide receptor radionuclide therapy (PRRT), targeting SSTR2, has shown cytoreductive potential and prolonged disease progression-free survival (PFS) in patients with unresectable metastatic disease (17, 18). Although PRRT extends PFS, about 15–30% of patients with advanced well-differentiated GEP-NETs progress during treatment or six months to one year after PRRT (19–22). Future optimization of PRRT will depend on improved patient stratification (23, 24). Currently, molecular functional imaging with positron emission computed tomography (PET/CT) is used in PanNETs management and updated European Neuroendocrine Society (ENETS) consensus on Radiological, Nuclear Medicine & Hybrid Imaging recommended ^68^Gallium (Ga)-DOTA-somatostatin analog-PET/CT for tumor staging, preoperative imaging, and re-staging. PET with ^68^Ga-DOTA-somatostatin analogs reveals SSTRs over-expressing lesions. Although the sensitivity and specificity of SSTR2-specific ^68^Ga-DOTATOC-PET/CT has been proven, its clinical utility is hampered by heterogeneous SSTR2 expression. Indeed, heterogeneous to low levels of SSTR2 expression challenge ^68^Ga-DOTA-somatostatin analogs-PET/CT sensitivity (25), thus eligibility to SSTR2-based therapies, such as PRRT (15, 16). Despite high SSTR2 expression can be considered an appropriate predictor of response to PRRT, PET/CT scan with ^68^Ga-DOTA-somatostatin analogs alone does not represent a prognostic parameter in terms of PFS (25). Besides Somatostatin Receptor based functional Imaging (SRI), 2-deoxy-2-[fluorine-18] fluoro-D-glucose (^18^F-FDG) PET/CT is also recommended for high grade well-differentiated GEP-NETs, especially for PanNETs, which generally display higher glucose metabolism and aggressiveness. ^18^F-FDG positive lesions are associated with worse prognosis, aggressive tumor behavior and resistance to PRRT even in low grade, well-differentiated GEP-NETs (26) while well-differentiated SINETs typically show less pronounced uptake of radiolabeled glucose and lower sensitivity at ^18^F-FDG-PET/CT scan (27).

Despite functional imaging with ^68^Ga-DOTA-somatostatin analogs and ^18^F-FDG-PET/CT are widely used for PRRT eligibility and for prognostication, about 60% of patients with advanced GEP-NET do not respond to PRRT. There is a clinical need for measurable and monitorable prognostic and predictive biomarkers which can supplement grade, stage, and imaging, improving patient stratification to address more tailored treatments for PanNETs (3, 28).

Blood biomarkers are easy to assess, minimally invasive, reproducible and can be used for real-time quantitative monitoring. Moreover, liquid markers overcome limitations of tissue specific information, providing a real-time snapshot of the disease and of tumor metabolism. Circulating miRNAs are well-established biomarkers for disease detection and monitoring. Exosome-encapsulated circulating miRNAs can be delivered to target cells promoting paracrine signaling and represent the source of choice for miRNAs in terms of quantity, quality, and stability (29). We sought to identify circulating miRNAs correlating with ^18^F-FDG functional imaging and with aggressive tumor metabolism in GEP-NETs, evaluating their accuracy as prognostic biomarkers to improve the clinical management of PanNET patients.

## Methods

### Study design

Plasma was collected from well-differentiated G1, G2 and G3 GEP-NET patients (n=24) who were mandatory free from any treatments, from at least one month before 177Lu-DOTATATE cycles start and whole miRNome Next Generation Sequencing (NGS) performed. MiRNA differential expression analysis between ^18^F-FDG PET/CT positive (n=12) and negative (n=12) patients of the screening set (n=24) was conducted. Since increased glucose uptake and higher prognostic power of ^18^F-FDG PET/CT in PanNETs is described, in order to identify disease-specific metabolic signatures, bioinformatic analysis was applied to the screening set (n=24) considering PanNETs (n=6) and SINETs (n=18), separately. MiRNA differential expression of ^18^F-FDG PET/CT positive and negative PanNET and SINET separated subsets was performed. Differentially expressed miRNAs between ^18^F-FDG PET/CT positive and negative patients were validated by RT/qPCR in plasma samples from two different validation cohorts of PanNETs (n=38) and SINETs (n=30). Additional comparison of miRNAs expression level was performed including healthy donors (n=17). Subsequently, we focused on the PanNET cohort only for further analyses. Assessment of validated miRNAs as potential independent predictors of PFS and of OS, alone or together with other canonical clinical, pathological and imaging features in PanNETs was evaluated. On the basis of the results obtained in plasma from the significant correlations with clinical and pathological features of PanNETs, we lately focused on the best candidate miRNA, hsa-miR-5096, to explore its relationship with ^68^Ga-DOTATOC-PET/CT SUV_max_ and SSTR2 expression. MiRNA and SSTR2 relative expression was then detected and quantified on a pilot independent cohort of PanNET tissue specimens (n=9) in order to assess if the inverse correlation with ^68^Ga-DOTATOC-PET/CT SUV_max_ (mirroring SSTR2 expression), observed in plasma, could be retrieved also on the tumor tissue. The computational output analysis quantified the relative miRNA SSTR2 expression, at the single cell level providing the rationale to explore the mechanism behind this inverse correlation *in vitro* on PanNET preclinical models.

### Clinical information on GEP-NET patients and healthy donors

From October 2016 to September 2019, patients with well-differentiated, advanced, metastatic, inoperable histologically or cytologically confirmed G1, G2 and G3 GEP-NET, were enrolled in the non-randomized LUX (NCT02736500) and LUNET (NCT02489604) clinical trials. Each patient enrolled in clinical trials was > = 18 years old, both genders and presented with a RECIST based progressive disease (PD). Patients displayed appropriate hematological, liver, and renal parameters (hemoglobin > = 10 g/dL; absolute neutrophil count (ANC) > = 1.5 x 109/L; platelets > = 100 x 109/L; bilirubin ≤ 1.5 X UNL (upper normal limit), ALT < 2.5 X UNL (< 5 X UNL in presence of liver metastases), creatinine < 2 mg/dL) were enrolled. Eligible patients did not receive other PRRT, radio- or chemotherapy, including capecitabine from one month before to two months after the completion of 177Lu-DOTATATE (alone or in combination) cycles. Patients were naive from previous radionuclide treatments with radiopeptides (e.g., ^111^Inpentetreotide, ^90^Y-DOTATOC) or other radiopharmaceuticals (e.g., ^131^I-MIBG, ^131^I). Each included patient presented measurable disease to conventional imaging evaluation (CT or MRI). SSTR-2 evaluation at time of enrollment was performed by ^68^Ga-DOTATOC-PET/CT and all included patients displayed ^68^Ga-DOTATOC-PET/CT uptake (SUV_max_) > 9, thus considered positive and mirroring SSTR2 expression. Patients enrolled in LUX clinical trials displayed ^18^F-FDG SUV_max_ > 2.5 at PET-CT scan, while patients in LUNET were negative. For this biological retrospective study, the screening cohort of GEP-NETs (n=24) and validation cohorts of PanNETs (n=38) and SINETs (n=30) included patients with well-differentiated G1, G2 and G3 advanced metastatic disease prior to **^177^** Lu-DOTATATE PRRT with a median follow up of 23.3 months (range: 6.5-60.9). **Supplementary Table S1** for patients and healthy demographic, clinical and pathological features. All patients provided a signed informed consent for the blood withdrawal, prior to ^177^Lu-DOTATATE PRRT and downstream genomic analysis. This study was approved by the local ethical committee (CEROM), approval no. 6711/5.1/2016, and performed according to Good Clinical Practice standards and the Declaration of Helsinki. The study protocol was amended to allow the collection of histologically confirmed G1, G2 and G3 PanNETs specimens to evaluate the hsa-miR-5096 and SSTR2 relative expression at the tissue level.

### Plasma specimen’s collection

Blood samples were collected by venipuncture at baseline, prior to ^177^Lu-DOTATATE PRRT. Blood was collected in a 3 mL K3-EDTA collection sterile vessel. Whole blood was centrifuged at 2500g for 10 minutes at room temperatures to obtain *platelet free* plasma. Plasma was carefully transferred into new 15 mL conical tubes (Falcon ™) for a second centrifugation at 2500 × *g* for 10 min to remove further cellular debris. At least 1 ml of supernatant was collected and stored at -80°C until required. Samples from the healthy donors cohort were and treated as well and collected at the same time to blood withdrawal to minimize differences in plasma composition.

### Small - RNA *exosome - enriched* fraction precipitation

Thawed, frozen plasma samples were precipitated using Exoquick™, SCBI according to the manufacturer’s protocol to obtain exosome-enriched fraction small-RNAs. Exoquick™, SCBI allows the precipitation of 20-100 nm vesicles and to extract their content. The pellet containing exosome-enriched fraction RNAs was resuspended in 200 ul of sterile PBS (1X). Qiazol™ was added to provide cryopreservation and lysis for exosome associated miRNA extraction.

Small RNAs, including miRNAs, were isolated with miRNeasy serum/plasma kit (Qiagen Cat No./ID: 217184) according to the manufacturer’s protocol. One mL of plasma per sample was used as input for the small-RNAs extraction. Small-RNAs isolated from the exome-enriched fraction, were eluted in 56 μL of RNase-free water.

### Whole miRNome NGS profiling and pipeline of analysis

Plasma specimens from the screening cohort of 24 GEP-NET patients were profiled for whole miRNome NGS. Small RNA transcripts were converted into barcoded cDNA libraries. Library preparation was created with the NEBNext Multiplex Small RNA Library Prep Set for Illumina (New England BioLabs Inc., USA). Libraries from each sample were pooled together and run on Illumina NextSeq 550 platform, 75 cycles (Illumina, USA). The obtained BCL Files were converted to FASTQ Files and data quality was assessed by FastQC software (RRID: SCR_014583). Secondary analysis was performed using docker4seq package [docker4seq, RRID: SCR_017006] (30, 31). Specifically, reads shorter than 14 nucleotides were discarded from the analysis; the remaining reads were trimmed from the adapter sequences using Cutadapt software(RRID : SCR_011841; https://journal.embnet.org/index.php/embnetjournal/article/view/200).

The trimmed reads were mapped against the precursor miRNA sequences downloaded from miRBase (Release 21) by the Shrimp algorithm (32). The counts matrix generated by the mapping was used as input for DESeq2 (RRID : SCR_000154) Bioconductor’s package [RRID : SCR_006442 (33);, to identify differentially expressed miRNAs between the ^18^F-FDG/PET positive and negative groups. Endogenous controls for RT/qPCR were selected from the NGS data by considering the following criteria for each raw data: at least 5 reads for each sample and a log_2_ standard deviation value < 16.

Bioinformatic pipeline of analysis encompassed principal components analysis (PCA) to exclude samples with poor number of reads. Only miRNA displaying at least one read in one of the samples were considered. MiRNAs differential expression analysis between ^18^F-FDG/PET/CT positive and negative GEP-NET patients was conducted considering Log_2_FC > = 1 and adj. p-value < 0.1. Second step correction was applied to exclude sample biases due to the tumor site of origin.

### Quantitative PCR validation of differentially expressed candidate miRNAs

Independent technical validation of candidate miRNAs was conducted by RT/qPCR in two distinct well-differentiated validation cohorts of 38 PanNETs and 30 SINETs. cDNAs from frozen and thawed RNA were obtained on C1000 Touch Thermal Cycler (Bio rad™, Hercules, CA, USA); using TaqMan™ MicroRNA Reverse Transcription Kit (Applied Biosystems™; Foster city, CA, USA. Cat No./ID: 4366596), cycling conditions were set according to the manufacturer’s protocol. TaqMan™ MicroRNA Reverse Transcription protocol was optimized multiplexing the TaqMan^®^ miRNA Assay’s primers for the following targets: hsa-miR-3133, hsa-miR-4311, hsa-miR-5096, hsa-let-7i-3p normalized with hsa-miR-30d as reference housekeeping miRNA (multiplexing group 1); hsa-miR-519c-3p, hsa-miR-582-3, hsa-miR-3614-5p, hsa-miR-1246 and hsa-miR-423-3p as reference housekeeping miRNA (multiplexing group 2).

Universal Master Mix without UNG and TaqMan™ miRNA Assay specific probes, for each target miRNA were used according to the manufacturer’s protocol (Applied Biosystems™, Foster city, CA, USA. Cat No./ID: 4440040). RT/qPCR analysis was conducted using Applied Biosystems™ 7500 Real-Time PCR Systems (Applied Biosystems™; Cat No./ID: 4351104).

Expression level of single target miRNAs was normalized to hsa-miR-30d and the fold enrichment was obtained by means of the 2^-ΔCT^ method, for the corresponding sample. In addition, *“Predictors”* (P1, P2, P3 and P) were created as the product of fold enrichments (2^-ΔCT^) of single miRNAs, to improve single targets and prognostic power (see **Supplementary Table**, **Figure S2**).

### miR-protein *in situ* detection

A novel semi-automated miR-protein *in situ* staining protocol was developed for the simultaneous detection of hsa-miR-5096 and SSTR2 protein expression.MiRCURY LNA miRNA Detection probe for hsa-miR-5096, U6 small nuclear, *positive control* probe (Qiagen, Valencia, CA; Cat No./ID: 99002-15) and the scramble negative control probe (Qiagen, Valencia, CA Cat No./ID: 99004-15), were used. Each probe was labeled 5’3’DIG. Before starting, double-DIG-LNA probes were denatured by heating (90°C for 4 min) and then diluted to 50 nM in the ISH buffer (miRCURY LNA miRNA ISH Buffer Set-FFPE).The first phase (tissue preparation, permeabilization and hybridization) has been performed in manual mode according to the miRCURY LNA miRNA detection probe protocol, while the second phase (signal detection) is automated using the Ventana BenchMark ULTRA platform (Ventana Medical Systems,Tucson, Arizona, USA). The automated protocol includes endogenous peroxidase blocking, casein blocking (16 min), incubation (37°C for 1 h) with primary prediluted mouse anti-DIG antibody (Ventana Medical Systems), to reveal the miR signal detected with OptiviewDAB Detection Kit (Ventana Medical Systems), consisting of HQ Universal Linker incubation (for 12 min), HRP Multimer incubation (for 12 min), and amplified with the Optiview DAB Amplification Kit (12 min). The revelation of SSTR2 protein expression was performed straight forward on Ventana BenchMark ULTRA, after cell conditioning with ULTRA CC1 (Ventana Medical Systems) for 24 min and casein blocking, using the antibody anti-SSTR2 (UMB1-C Terminal-ab134152-Abcam) in Ventana antibody diluent, incubated (37°C for 1 h), detected with Ultraview Universal Alkaline Phosphatase Red Detection Kit (Ventana Medical Systems). Finally, slides were counterstained for 8 minutes with Haematoxylin II (Ventana Medical Systems) and for 8 minutes with Bluing Reagent (Ventana Medical Systems), washed in tap water with soap to remove the liquid coverslip, dehydrated in the stove and mounted with xylene and EUKITT mounting medium (Sigma-Aldrich, Merck KGaA, Darmstadt, Germany). Specifically, this protocol reveals as first marker the miR in brown by using an anti-DIG antibody followed by the protein detection in red with the anti-SSTR2 antibody. Labeling with digoxigenin (DIG) allows miR- staining stability after double immunohistochemical rounds performed on the automated Ventana platforms. Additionally, our approach avoids antigen retrieval which typically occurs when immunohistochemistry is performed prior to ISH. For this purpose, we compared the results obtained from the single IHC for SSTR2 expression with those obtained with the miR-protein protocol and we assessed that there were no differences in terms of protein expression (Source Data not shown, see Availability of data and materials section for data repository). IHC whole slides images were acquired with the high-resolution slide scanner Aperio CS2 using the focus-ISH algorithm with a 40x magnification, which provides scanned images with the accuracy and resolution required for ISH.

### *AND-Tool* software interface development

To analyze the marker expression of the single nuclei in the histological samples, we designed a user-friendly open-source Graphical User Interface (GUI) requiring a minimal user interaction. The GUI has been named *Analysis Nuclei DAB (AND)-Tool* and it allows to automatically segment the nuclei and extract intensity/morphological features at the single-nuclei level. The AND-Tool was created using Matlab (The MathWorks, Inc., Massachusetts, USA). Source code, standalone executable version, documentation, and sample images are available for download from: https://sourceforge.net/p/andtool/. First, all the acquired RGB images were corrected for uneven illumination by subtracting the background estimated with the standard ImageJ/Fiji rolling ball algorithm. Then, the RGB images were unmixed using the Color Deconvolution ImageJ/Fiji plugin imposing the “FastRed-FastBlue-DAB” modality (34). The FastRed channel was used to subdivide the field of view into three distinguished types of regions of interest (ROIs, i.e., “dark-red”, “light-pink” and “white” ROIs) according to the local intensity and two fixed thresholds (hereafter named Th1 and Th2, with Th1 lower than Th2), manually defined from the user just once for all the images to be analyzed. The “dark-red” ROIs are those regions with intensity values of the FastRed channel between 0 and Th1; the “light-pink” ROIs, with intensity values between Th1 and Th2; the “white” ROIs, with intensity values between Th2 and 255. Nuclei have been detected using the FastBlue and the DAB channels. To detect the nuclei, we used an intensity-based k-mean classifier automatically subdividing the single channels into three regions: white background, weak cytoplasmic signal, and nuclear signal. The standard watershed segmentation algorithm was then used to analyze the nuclear signal and split touching objects to proceed in a single-nuclei analysis. Objects with size not compliant with that of a nucleus were filtered out to compute the masks of the real nuclei. Single-nuclei intensity/morphological features and region-based statistics were computed using the intensity maps created by subdividing the sample areas in dark-red, light-pink and white ROIs. Two types of nuclei have been considered: the ones positive for the DAB staining, and the ones positive for the FastBlue staining but not positive for DAB (see **Supplementary File S2** for software analysis pipeline and manual). AND-Tool software analysis considered 10 fields per sample. The software was designed to identify three different levels of SSTR2 expressing nuclei: high as “dark-red” mask (identified by the software in the intensity range 0-Th1, with the threshold Th1: 100); intermediate as “light-pink” mask (identified by the software in the intensity range Th1-Th2, with the threshold Th2: 190) and the negative areas as “white” mask (identified by the software in the intensity range Th2-255, with 255 being the maximum value of intensity in the 8-bit gray-level conversion). AND-Tool was able to contemporary recognize areas with miR positive nuclei as DAB channel positivity. Correlation analysis has been conducted plotting the average percentage of hsa-miR-5096 positive nuclei on overall analyzed cells in different SSTR2 expression areas. Spearman test was applied to determine r2 and p value.

### Cell culture

Hsa-miR-5096 and SSTR2 expression was assessed in NT-3, BON-1 and QGP-1 cell lines (RRID: CVCL_VG81; CVCL_3985; CVCL_3143). NT-3 cells were cultivated in culture dishes coated with collagen type IV from Human Placenta (Sigma-Aldrich, Homefield Road, Haverhill, UK; Cat No./ID: 27663) in RPMI medium supplemented with 10% FCS, 1% penicillin/streptomycin and L-glutammine, 15mM HEPES both with EGF (20 ng/mL; PreproTech, Rocky Hill, New Jersey), and FGF2 (10 ng/mL; PreproTech, Rocky Hill, New Jersey) and without growth factors (bFGF; EGF) (35). BON-1 and QGP-1 were cultivated in culture dishes in DMEM high glucose and RPMI medium respectively, supplemented with 10% FCS, 1% penicillin/streptomycin and L-glutammine, 15mMHEPES.

### Hsa-miR-5096 mimic and inhibitor treatment

To evaluate SSTR2 downmodulation in NT-3 cell lines, 3 × 10^5^ cells were plated into 6-well dishes coated with collagen type IV from Human Placenta. After 24 hours, 15 and 30 pmol of hsa-miR-5096 miRCURY LNA miRNA Mimic and Scramble (Qiagen, Valencia, CA) were transfected using RNAiMAX transfection reagent (Invitrogen^®^, Carlsbad, CA, USA) according to the manufacturer’s instructions. Conversely, to evaluate SSTR2 up-modulation in NT-3 cell lines, 5 × 10^5^ cells were plated into 12-well dishes coated with collagen type IV from Human Placenta; while 1.75 and 2.5× 10^5^ of BON-1 and QGP-1 were plated into standard 12-well dishes, respectively. Then, 100 nM pmol of hsa-miR-5096 miRCURY LNA miRNA Inhibitor and Scramble (Qiagen, Valencia, CA) were transfected using RNAiMAX transfection reagent (Invitrogen^®^, Carlsbad, CA, USA) according to the manufacturer’s instructions. Cells and culture medium were collected after 48 and 72h transfection. A Fixed volume of 350 ul of Trizol^®^ reagent has been added to dried pellets and miRNeasy Mini Kit 50 (Qiagen, Valencia, CA Cat No./ID: 217004) was used for RNA extraction to quantify hsa-miR-5096 and SSTR2 RNA levels. *SSTR2* expression in NT-3 cell lines was assessed by RT/qPCR, after 48h and 72h mimic and inhibitor transfection. Expression values were expressed as (Ct) values normalized to the housekeeping gene HPRT (2^−ΔCT^ method), and then represented in terms of percentage of expression.

### Immunofluorescence

To evaluate SSTR-2 protein expression after the inhibition of hsa-miR-5096 *via* miRCURY LNA, we performed immunofluorescence staining on QGP-1 cells grown and transfected on coverslip slides. QGP-1 cells were fixed for 10 minutes at 4° C with 10% Formalin, permeabilized with Tween-Triton 0.3% in PBS and blocked with 1% Bovine Serum (BSA) before incubation with the Human Somatostatin R2/SSTR2 PE-conjugated Mouse IgG2A Antibody (Clone # 402038) for 2h at room temperature. Dapi staining was used to counterstain the nucleus.

### Statistical analyses

Sample size (n=24) of the screening cohort GEP-NET was established on the basis of a desired 2-fold change, a sigma=0.5 from a previous knowledge, a power of 80%, an FDR (false discovery rate) of 0.05 and a proportions of non-differentially expressed genes of 98%, using ssize.twoSamp R package. In addition, balancing of patients bearing ^18^FDG-PET positive (n=12) and negative (n=12) was considered to avoid biases of the differential expression analysis. Sample size of the validation cohorts of PanNETs (n=38) and SINETs (n=30) was determined taking into account enrollment rate of LUX and LUNET clinical trials, the low prevalence and incidence of GEP-NET disease, NGS technical or stratification requirements, mutual subgrouping (FDG+; FDG-), presence of hemolytic samples and economic feasibility. Considering a drop-out rate of 10%, it was feasible to enroll at least 66 patients in the study. An additional cohort of 17 healthy donors balanced in terms of age, sex and time to blood withdrawal was considered for blood withdrawal and subsequent molecular comparison with PanNETs. The sample size of the healthy volunteer cohort was designed to be consistent with the patient’s population, considering that comparison with healthy volunteers was not the goal of the present study. Of note, as reported in the **Supplementary File S1** and **Supplementary Figure S1**, we ruled out age as a confounding factor for hsa-miR-5096 levels in both PanNETs and healthy donors. Categorical data were expressed as absolute numbers and percentage, while continuous variables were shown as median and range. Normality of distribution of continuous data was assessed through the Shapiro-Wilk test. MiRNAs normalized median expression level (2^-ΔCT^) were compared between ^18^F-FDG PET/CT positive and negative groups. Wilcoxon and Mann-Whitney test, chi-square tests were applied respectively for continuous data and categorical data. For comparison between three groups Kruskal-Wallis test was used and Dunn test was used for *post-hoc* comparisons.

The Receiver Operating Characteristic (ROC) curve, defined as a plot of sensitivity vs 1-specificity, was performed as evaluation of the performance of candidate miRNAs and their combination to predict PET positivity, 6-month (-mo) PFS and 12-mo OS. AUC (with 95% confidence - CI) was calculated as a common measure of accuracy and values range from 0.5 to 1.0: higher values are corresponding to a better performance of tested values. AUC values higher than 0.7 were considered as acceptable values. Roccomp STATA command was used to compare the ROC curves. For each curve, roccomp reports summary statistics and provides a test for the equality of the area under the curves, using an algorithm suggested by DeLong and Clarke-Pearson (36).

Cut-off with higher value of sensitivity and specificity was evaluated. OS was calculated as the time from date of start PRRT therapy to date of death or last follow-up visit, while PFS was calculated as the time from date of start PRRT therapy to date of progressive disease, death, or last follow-up visit. Alive patients were censored at last visit while patients without disease progression were censored at last tumor evaluation. Kaplan-Meier (KM) curves were used to estimate the survival function and the log-rank test was used to compare different subgroups in terms of OS or PFS. Median OS and median PFS were calculated, and 95% confidence intervals (95%CI) were reported. Univariable and multivariable Cox regression models were carried out with the explorative intent of evaluating the potentially independent clinical parameters associated with PFS and OS, including the miRNA of interest. These models have been evaluated for exploratory intent and should be validated on an enlarged cohort. Threshold for including variables in multivariable models for PFS was a p-value of 0.10. Further evaluation will be done regarding collinearity among potential independent factors. Analysis to explore imaging parameters didn’t consider any threshold. Outcome evaluation was performed using a complete case analysis, without any type of imputation. Transfection efficacy and statistical significance for *in vitro* experiments were assessed by parametric t-test, comparing expression median value (+/-SD). For comparison between three groups Kruskal-Wallis test was used and Dunn test was used for *post-hoc* comparisons.

All statistical analyses were performed using Stata/SE version 15.1 for Windows (StataCorpLP, College Station, TX, USA). Time ROC R package was used to plot time-dependent AUC curve and 95% confidence interval.

## Results

### Circulating exosomal miRNA-signature as a surrogate prognostic biomarker for ^18^F-FDG PET/CT status in PanNET patients

**Figure 1:**
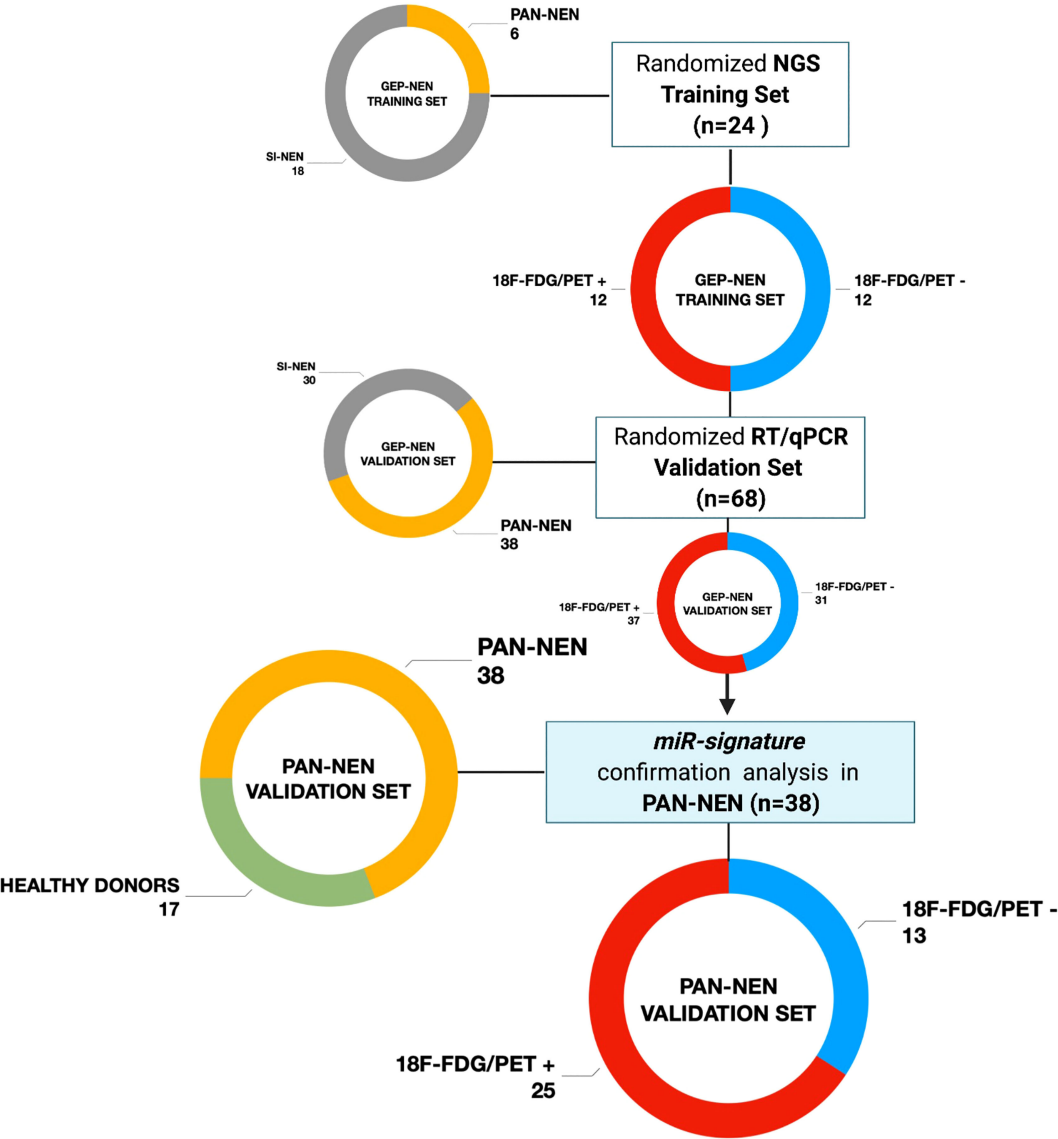
Study design flow-chart Patients population schematic view summarizing the study design flow-chart. GEP-NET (n=68): Gastro-Entero-Pancreatic Neuroendocrine Tumor; PanNET (n=38): Pancreatic Neuroendocrine Tumor; SI-NET (n=30): Ileal Neuroendocrine Neoplasm; 18F-FDG/PET + (n=25): positive (SUV_max_ > 2.5); 18F-FDG/PET - (n=13): negative (SUV_max_ < 2.5). Created with BioRender.com.

**Figure 2:**
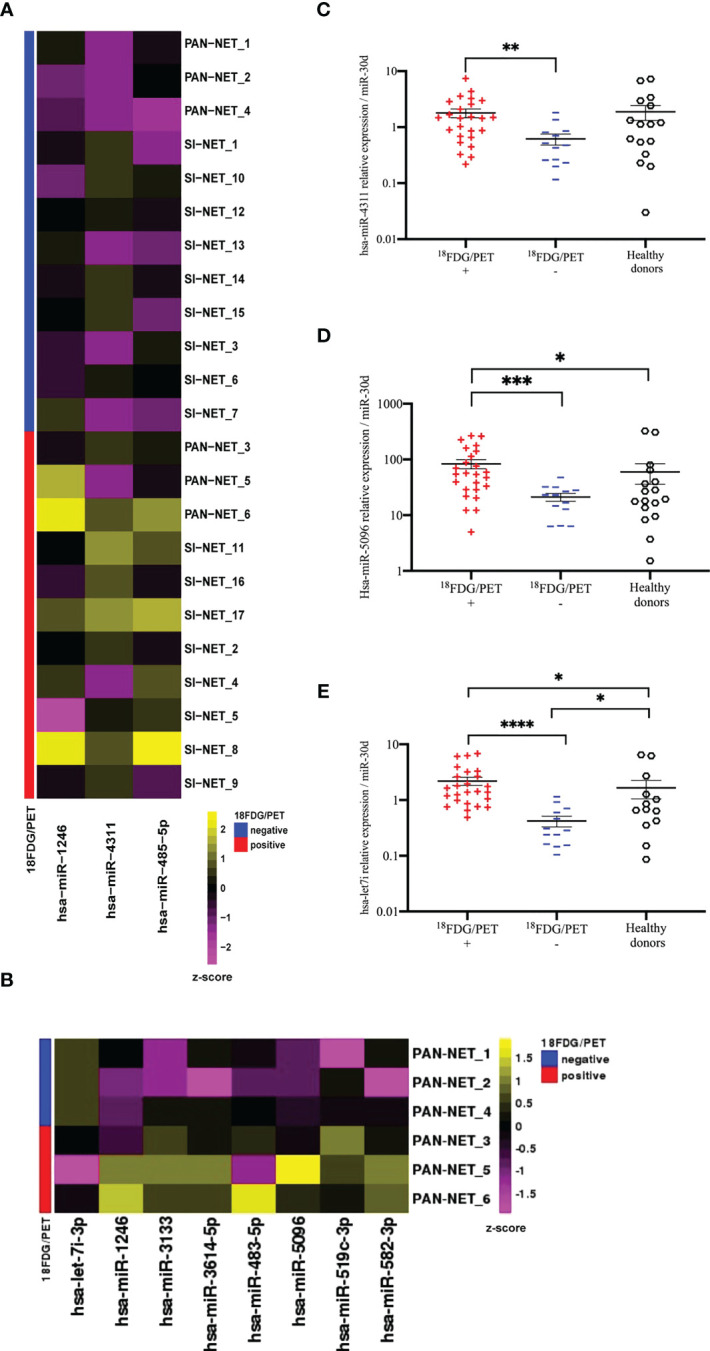
NGS analysis of circulating exosomal miRNAs in GEP-NETs revealed a metabolic signature in PanNET patients **(A)** The heat-map shows the expression pattern of three differentially expressed miRNAs between 18F-FDG/PET positive (n=12) and negative (n=12) GEP-NET training set (n=24). Each column represents a single miRNA while each row represents a single sample. The heat-map was obtained with the DeSeq2 package on regularized logarithm transformed counts. Color code is reported above the heat-map. **(B)** The heat-map shows log2 normalized counts for all significant DEGs selected from comparison between 18F-FDG/PET positive (n=3) and negative (n=3) PAN-NET training subset. Yellow colors indicate over-represented and purple colors under-represented genes in comparison to the corresponding PAN-NET 18F-FDG/PET negative. **(C–E)** Deregulated miRNAs between 18F-FDG/PET positive (n=25) and negative (n=13) PanNETs validation sets: **(C)** hsa-miR-4311 **(D)** hsa-miR-5096 and **(E)** hsa-let-7i-3p. Hsa-miR-30d was selected from NGS profiling as an endogenous control. Results are presented as mean ± SD (*p-value<0.05; **p-value<0.01; ***p-value<0.001; ****p-value<0.0001). Wilcoxon and Mann-Whitney test, chi-square tests were applied respectively for continuous data and categorical data. For comparison between three groups Kruskall-Wallis test was used and Dunn test was used for *post-hoc* comparisons.

In order to identify circulating prognostic and measurable miRNAs associated with ^18^F-FDG PET/CT positivity in GEP-NET patients, we evaluated plasma specimens from advanced metastatic GEP-NETs, comparing ^18^F-FDG PET/CT positive and negative patients. In the screening step, plasma from well-differentiated G1, G2 and G3 GEP-NETs (n=24) was collected at baseline, prior to 177Lu-DOTATATE PRRT and whole miRNome using Next Generation Sequencing (NGS) was performed (**Figure 1**). Profiling was conducted on a screening set of well-differentiated (G1, G2 and G3) GEP-NETs comparing ^18^F**-**FDG-PET/CT positive (n=12) and negative (n=12) tumors. Principal component analysis (PCA) excluded one out of 24 samples due to poor number of reads. NGS analysis identified 2588 miRNAs. Of those, 2474 miRNAs displayed at least one read in one of the samples analyzed. The bioinformatic analysis revealed hsa-miR-1246, hsa-miR-4311 and hsa-miR-485-5p as differentially expressed miRNAs (Log_2_FC> = 1; adj. p-value: < 0.1) between ^18^F-FDG PET/CT positive and negative GEP-NET patients (**Figure 2A**). Second step analysis considered SINETs and PanNETs separately, to assess if disease specific signatures exist, and revealed 8 miRNAs (hsa-miR-1246; hsa-miR-5096; hsa-let-7i-3p; hsa-miR-3133; hsa-miR-3614-5p; hsa-miR-483-5p; hsa-miR-519c-3p; hsa-miR-582-3p) as differentially expressed between ^18^F-FDG PET/CT positive and negative PanNETs patients (**Figure 2B**). Conversely, no miRNA correlated with ^18^F-FDG PET/CT status in the SINET subset (data not shown). Finally, hsa-miR-30d emerged to be the miR- with the lowest standard deviation in the number of normalized reads among all case series (Coefficent of Variantion<0.05), thus in light of its stability it was selected as endogenous reference for all the comparisons. Altogether a group of 10 non redundant miRNAs were considered for validation by RT/qPCR in SINETs (n=30) and PanNETs (n=38) cohorts, separately. Three circulating miRNAs (hsa-miR-4311, p-value:<0.001; hsa-miR-5096, p-value:<0.0001; hsa-let-7i-3p, p-value:<0.00001) significantly correlated with ^18^F-FDG-PET/CT status in the PanNETs subgroup only (**Figures 2C–E**). Fold change expression values for the three single miRNAs were then combined into “*Predictors*” to test their combined prognostic power. “*Predictors*” were mathematically built, multiplying the fold enrichment value obtained for the single miRNAs in different combinations. We calculated 3 binary “*Predictors*” by combining, hsa-miR-4311*hsa-let-7i-3p (P1), hsa-miR-5096-5p*hsa-let-7i-3p (P2), hsa-miR-4311*hsa-miR-5096-5p (P3), and 1 ternary predictor which combines, hsa-miR-4311*hsa-miR-5096-5p*hsa-let-7i-3p fold changes together (P). All “*Predictors*” significantly correlated with 18F-FDG-PET/CT. Fold change expression values for the three single miRNAs and combined “*Predictors*” are reported in **Supplementary Table S2** and **Supplementary Figure S2**. Statistical analysis, according to 18F-FDG PET/CT excluded age contribution to miR-signature predictivity (**Supplementary Figure S1**, **Supplementary File S1**, **Supplementary Table S1**).

**Figure 3:**
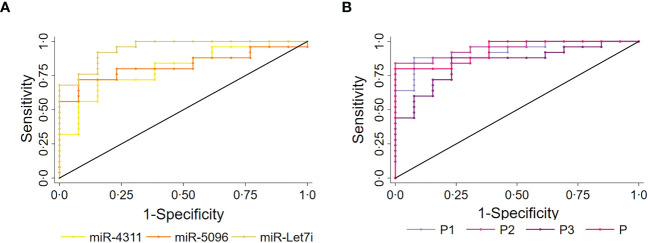
Circulating miRNAs signature predicts 18F-FDG/PET positivity in PanNET patients. Performance of the circulating signature in predicting 18F-FDG/PET outcome in PanNET validation set (n=38). **(A)** ROC curve of the single miRNAs: hsa-miR-4311; hsa-miR-5096: hsa-miR-5096; miR-let7i: hsa-let-7i-3p. Roccomp plots the ROC curves on the same graph. **(B)** ROC curve of combined predictors, with significantly high AUCs. miR-4311: has-miR-4311; miR-5096: has-miR-5096; miR-let7i: hsa-let-7i-3p. DeLong, and Clarke-Pearson (36) algorithm was applied to provide a test for the equality of the area under the curves.

The predictive power of single miRNAs in relation to 18F-FDG PET/CT positivity was evaluated. ROC analysis revealed that higher circulating expression levels of hsa-miR-4311, hsa-miR-5096 and hsa-let-7i-3p alone or combined into “*Predictors*” can predict ^18^F-FDG PET/CT positive outcome with significant AUCs between 0.81 and 0.95, with P2 (hsa-miR-5096-5p*hsa-let-7i-3p) showing the best performances (**Figure 3**, **Table 1**; **Supplementary Table S3**).

**Table 1:** Single miRNAs significant AUC values, sensitivity and specificity of ^18^FDG/PET prediction in PAN-NEN patients.

miRNA	AUC (95%CI)	Proposed cut-offs	Sens. (%)	Spec. (%)
**hsa-miR-4311-5p**	0.8062 (0.66-0.94)	0.85	72	85
**hsa-miR-5096**	0.8246 (0.69-0.95)	70	40	100
**hsa-Let7i-3p**	0.9477 (0.88-1.99)	0.72	92	85

AUC, Area Under the Curve; Sens.(%), sensitivity percentage; Spec.(%), specificity percentage; C.I, Confidence interval.

Overall, three prognostic miRNAs were retrieved from the exosomal fraction of plasma in PanNET patients (n=38) and were found associated with 18F-FDG PET/CT positivity. We showed that their fold change can be combined into oligo analytes “*Predictors*” with similar predictivity in our Pan-NET cohort. However, we hypothesize that the use of oligo analytes biomarkers over single miRNAs should grant higher accuracy as the number of patients assayed will increase. The statistical comparison among the AUC values of the single miRNAs and the “*Predictors*” are reported in **Supplementary Table S3**.

Given this, further analyses were conducted, revealing hsa-miR-5096 alone or in combination as best candidate prognostic biomarkers for clinical usage, showing the best performances in predicting different endpoints.

### Circulating exosomal hsa-miR-5096 is a potential independent predictor of survival for PanNETs

**Figure 4:**
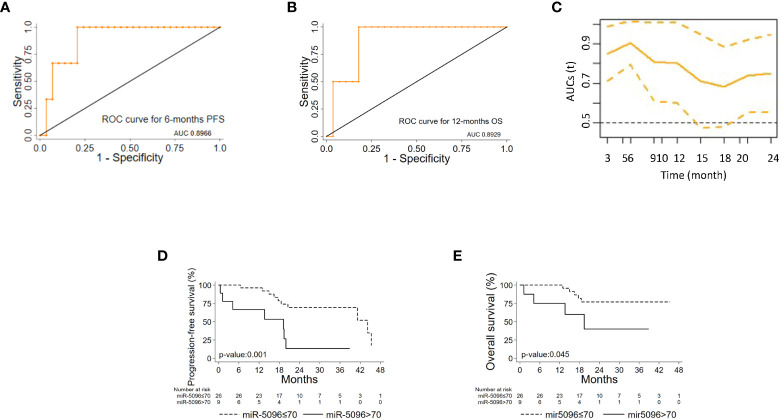
hsa-mir-5096 can predict PFS and 12-mo OS in PanNET patients. Performance of circulating hsa-miR-5096 in predicting 6-mo PFS and 12-mo OS in PanNET patients treated with 177Lu-DOTATATE (n=38). ROC curve analysis **(A, B)** of: **(A)** hsa-miR-5096 for 6-mo PFS; **(B)** hsa-mir-5096 for 12-month OS. **(C)** Time dependent AUC curve (95% C.I) for hsa-miR-5096 prediction of 3–24-month PFS; TimeROC package in R software was used to provide an estimation of time-dependent ROC curve and time dependent AUC in the presence of censored data. Kaplan–Meier analysis **(D, E)** of: **(D)** PFS by hsa-miR-5096 in PanNET patients, including at risk patients for each stratum; **(E)** OS by hsa-mir-5096 in PanNET patients, including at risk patients for each stratum.

ROC analysis of single miRNAs (at baseline) was conducted for PanNET patient’s subset (n=38) that were subsequently treated with ^177^Lu-DOTATATE based PRRT and showed that hsa-miR-5096 best predicts 6-mo PFS (AUC: 0.8966; 95% CI: 0.76 - 1.00; **Figure 4A**) and 12-month OS (AUC: 0.8929; 95% CI: 0.72-1.00; **Figure 4B**). Time dependent (range: 3 - 24 months) ROC curve analysis for PFS showed that hsa-miR-5096 maintains prognostic AUC values up to 24 months (**Figure 4C**; TimeROC package in R software was used to provide an estimation of time-dependent ROC curve and the associated time dependent AUC in the presence of censored data). Furthermore, circulating hsa-miR-5096 expression level (cut-off: 70) distinguished PanNET patients with poor prognosis from responders to PRRT for both PFS (p-value:<0.001; **Figure 4D**) and OS (p-value:< 0.05; **Figure 4E**).

**Figure 5:**
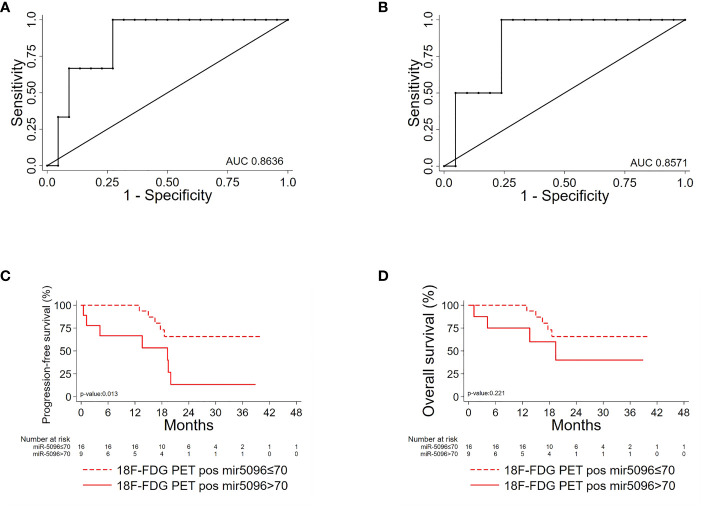
Hsa-miR-5096 can predict PFS and 12-mo OS in 18F-FDG/PET positive PanNET patients. Performance of circulating hsa-miR-5096 in predicting 6-mo PFS and 12-mo OS in 18F-FDG PET/CT positive (+) PanNET patients treated with 177Lu-DOTATATE (n=25). ROC curve analysis **(A, B)**: **(A)** hsa-miR-5096 for 6-mo PFS in 18F-FDG PET/CT positive (+) patients; **(B)** hsa-miR-5096 for 12-month OS in 18F-FDG PET/CT positive (+) patients. Kaplan–Meier analysis **(C, D)** of: **(C)** PFS by hsa-miR-5096, including at risk patients for each stratum; in 18F-FDG PET/CT positive subgroup; **(D)** OS by hsa-miR-5096 including at risk patients for each stratum in 18F-FDG PET/CT positive subgroup.

Importantly, hsa-miR-5096 emerged to be an accurate predictor of 6-mo PFS (AUC: 0.8636; 95% CI: 0.68-1.00; **Figure 5A**) in the subgroup of ^18^F-FDG PET/CT positive patients, characterized by more aggressive disease. Specifically, a cut-off of 70 resulted in 100% sensitivity and 68% specificity identifying a subset of patients that progress earlier and do not benefit from ^177^Lu-PRRT treatment (p-value:< 0.01; **Figure 5C**). It is worth noting that the LIU/Yuden *standard computed* cut-off shows equal performances to our *in-house* defined cut-off (70) for all investigated clinical endpoints, thus substantiating its robustness (data not shown). Finally, although hsa-mir-5096 represents an accurate predictor also for 12-mo OS in the ^18^F-FDG PET/CT positive subset (AUC: 0.8571; 95% CI: 0.63-1.00; **Figure 5B**), a cut-off of 70 could not significantly stratify ^18^F-FDG PET/CT positive patients for 12-mo OS predictions (p-value:0.22; **Figure 5D**). Significant AUCs, sensitivity and specificity values for 6-mo PFS and OS in overall and in ^18^F-FDG PET/CT positive PanNET patients are shown in **Table 2**.

**Table 2:** Hsa-miR-5096 significant AUC values, sensitivity and specificity of 6-month PFS and 12-months OS in PAN-NEN patients for ^18^FDG/PET positive subset predictions, treated with ^177^Lu-DOTATE PRRT.

Subset	Clinical Endpoint	AUC (95%CI)	cut-off	Sens. (%)	Spec. (%)
**PAN-NENs**	6-month PFS	0.8966 (0.76-1.00)	70	100	75
	12-months OS	0.8929 (0.72-1.00)	70	100	79
**^18^FDG/PET (+) PAN-NENs**	6-month PFS	0.8636 (0.68-1.00)	70	100	68
	12-months OS	0.8571 (0.63-1.00)	70	100	71

AUC, Area Under the Curve; Sens.(%), sensitivity percentage; Spec.(%), specificity percentage; C.I, Confidence interval. ^18^FDG/PET (+): ^18^FDG/PET positive PAN-NENs subgroup.

Moreover, considering overall PanNET patients, multivariable model for PFS including canonical clinico-pathological features showed that PanNET patients with hsa-miR-5096 > 70 had a higher risk of progressive disease, as shown in **Table 3A** (HR: 4.24, 95% CI:1.39-12.93, p-value: 0.011).

**Table 3A:** Univariable and multivariable Cox regression models for PFS including clinical parameters.

Variables	HR for univariable model (95%CI)	p-value	HR for multivariable model (95%CI)	p-value
Age (continuous variable)	0.97 (0.92-1.01)	0.093		
Gender: Female vs Male	2.80 (0.99-7.88)	0.051	3.01 (1.07-8.49)	0.037
Grading: G3 vs G1/G2 §	4.65 (1.21-17.81)	0.025	2.52 (0.61-10.33)	0.198
Ki67: >=20 vs <20 §	4.65 (1.21-17.81)	0.025		
Tumor burden: extensive or moderate vs limited	3.13 (0.68-14.37)	0.142		
Liver metastasis: Presence vs absence	3.38 (0.76-15.0)	0.109		
Bone metastasis: Presence vs absence	1.53 (0.52-4.49)	0.434		
Rotterdam Index: 4 vs 3	1.34 (0.46-3.91)	0.586		
hsa-miR-4311: >0.95 vs ≤0.95	1.80 (0.68-4.79)	0.235		
hsa-miR-5096: >70 vs ≤70	4.82 (1.66-14.02)	0.004	4.24 (1.39-12.93)	0.011
hsa-let-7i-3p: >1.38 vs ≤1.38	1.01 (0.38-2.68)	0.975		

HR: Hazard ratio; C.I: Confidence interval; § Ki67 and grading subgroup were collinear and in the multivariable model only grading was considered in multivariable model; # Specific cut off for 3-months PFS was calculated through ROC curve; @ P3AB not included in the multivariable model because it was a combination that includes hsa-miR-5096.

In addition, a second multivariable model considering the relationship of hsa-miR-5096 circulating levels with functional imaging parameters, further confirmed that patients with hsa-miR-5096 > 70 had a higher risk of progressive disease, as shown in **Table 3B** (HR: 5.98, 95% CI:1.28-24.86, p-value: 0.023). Univariate Cox regression model for OS was conducted with explorative intent and reported a HR of 3.53 for hsa-miR-5096 (95%CI:0.94-13.22, p-value:0.060). In summary, statistical analyses support hsa-miR-5096 as an accurate and independent predictor of PFS in PanNET patients and its increased level above 70 identify ^18^F-FDG-PET/CT positive patients with the poorest PFS after ^177^Lu-PRRT treatment.

**Table 3B:** Univariable and multivariable analysis of hsa-miR-5096 and imaging parameters for PFS.

Variables	HR for univariable model (95%CI)	p-value	HR for multivariable model (95%CI)	p-value
FDG PET result: Positive vs negative	1.48 (0.49-4.40)	0.478	3.51 (0.25-47.70)	0.346
SUV GA PET (Continuous variable)	0.99 (0.97-1.01)	0.645	1.01 (0.99-1.03)	0.313
hsa-miR-5096: >70 vs ≤70	4.82 (1.66-14.02)	0.004	5.98 (1.28-24.86)	0.023

### Hsa-miR-5096 expression inversely correlates with *SSTR2* expression in PanNET

**Figure 6:**
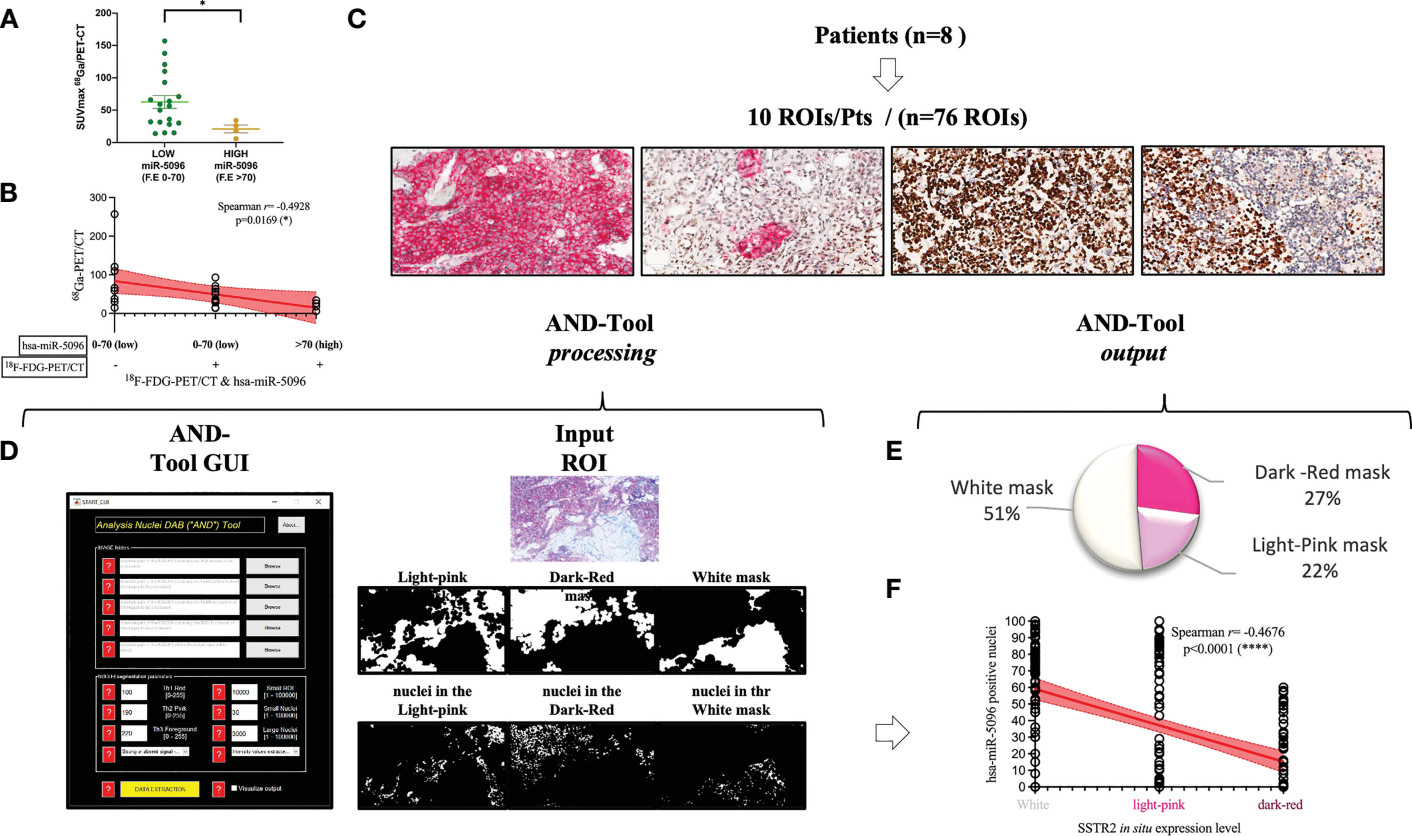
Hsa-miR-5096 overexpression inversely correlates with SSTR2 expression levels in PanNET patients. **(A)** correlation analysis of expression of circulating hsa-miR-5096 and 68Ga-PET SUV_max_ in plasma of PanNET patients (n=38 excluding 15 patients due to missing data on 68Ga-PET SUV_max_ value). Data comparison was conducted by means of Mann Whitney test (p: 0,04). **(B)** Correlation analysis (Spearman; p< 0,0169; r2: -0,4928) of 68Ga-PET/CT SUV_max_ and 18F-FDG-PET/CT positivity in patients displaying low and high levels of hsa-miR-5096 (cut-off:70) in plasma of PanNET patients. **(C)** Representative images of PanNET tumor heterogeneity: simultaneous detection of hsa-miR-5096 (DAB-BROWN) and SSTR2 protein (RED) in FFPE tumor tissue through our miR-protein protocol. **(D)** AND-Tool automated analysis on PanNET FFPE samples (n=8). On the left, a screenshot of the AND-Tool graphical user interface (GUI). On the right, an example of analysis with at the top part an input ROI. In the central part, light-pink, dark-red, and white masks, respectively. In the bottom part, segmented nuclei are subdivided for the different masks. **(E)** Illustrative diagram of overall analyzed pixels in terms of SSTR2 expression: frankly positive (27%), heterogeneous (22%) and negative (51%) expression areas; **(F)** Correlation analysis (Spearman; r=-0,4676; p<0,0001) of hsa-mir-5096 positive nuclei (%) in and SSTR2 expression level in FFPE PanNET specimens.

To further assess its clinical impact in PanNET management, the expression levels of the miRNAs of the signature were correlated with several clinico-pathological features, including ^68^Ga-DOTATOC PET/CT SUV_max_. Interestingly, increased expression levels of circulating hsa-miR-5096 (cut-off: 70) correlated with lower ^68^Ga-DOTATOC PET/CT SUV_max_ (Mann Whitney test, p-value < 0.05) in PanNET patients (**Figure 6A**). According with previous observation, a negative association of 68Ga-DOTATOC PET/CT SUV_max_ and ^18^F-FDG-PET/CT positivity in patients displaying low and high levels of hsa-miR-5096 (cut-off:70; Spearman: p< 0,0169; r2: -0,4928); was observed (**Figure 6B**). Since ^68^Ga-DOTATOC PET/CT SUV_max_ mirrors SSTR2 expression level in PanNET patients, the observed inverse correlation suggested that hsa-miR-5096 may be involved in SSTR2 regulation also at the tissue level. To confirm this hypothesis, a semi-automated immune-miRNA-ISH approach coupled with a dedicated pipeline of analysis (AND-Tool software) was set up and applied to detect and quantify hsa-miR-5096 and SSTR2 expression simultaneously on FFPE tumor tissue samples. Eight independent G1, G2 (n.5) and G3 (n.3) PanNET FFPE tumor tissue specimens were first reviewed by an expert pathologist for SSTR2 expression level. Two were negative, four were frankly positive (100%; 3+) and two displayed SSTR2 heterogeneous expression (**Figures 6C, D**). AND-Tool software analysis of n=8 PanNET FFPE samples, considering 10 ROIs (Regions Of Interest) per patient, 76 total ROIs (four ROIs drop-out due to presence of a tissue folding in one case sample), resulted in the extraction of 197847672 pixels, corresponding to an average value of 15186 ± 7547 analyzed cells per sample. Using AND-Tool software we extracted Dark-red, Light-pink, and White masks for each ROIs separately. Subsequently, we applied a pixel-based analyses of Dark-Red (SSTR2 positive), Light-Pink (SSTR2 low) and White (SSTR2 negative) masks showing 27% Dark-Red pixels, corresponding to the amount of frankly positive cells; 22% of Light-Pink pixels, corresponding to the amount of low expressing cells; and 51% White pixels of negative expression areas (**Figure 6E**). Correlation analysis confirmed a significant inverse association between the number of hsa-miR-5096 positive cells and SSTR2 expression level on PanNET tissue (Spearman; r=-0,4676; p<0,0001; **Figure 6F**). Importantly, areas with low/moderate SSTR2 expression, which also define patients eligible for PRRT, showed an intermediate frequency of hsa-miR-5096 positive nuclei. These observations agree with a mechanistic model where hsa-miR-5096 expressing cells can contribute to tumor heterogeneity and mosaicism through a paracrine SSTR2 interference which could hinder PanNET targeting and ineffective responses to PRRT. Those results show that hsa-miR-5096 is expressed by PanNET tumor cells and that, the inverse correlation between circulating hsa-miR-5096 levels and ^68^Ga-DOTATOC PET/CT SUV_max_ values, mirrors an interplay occurring also at the tissue level.

### Hsa-miR-5096 modulates SSTR2 expression

**Figure 7:**
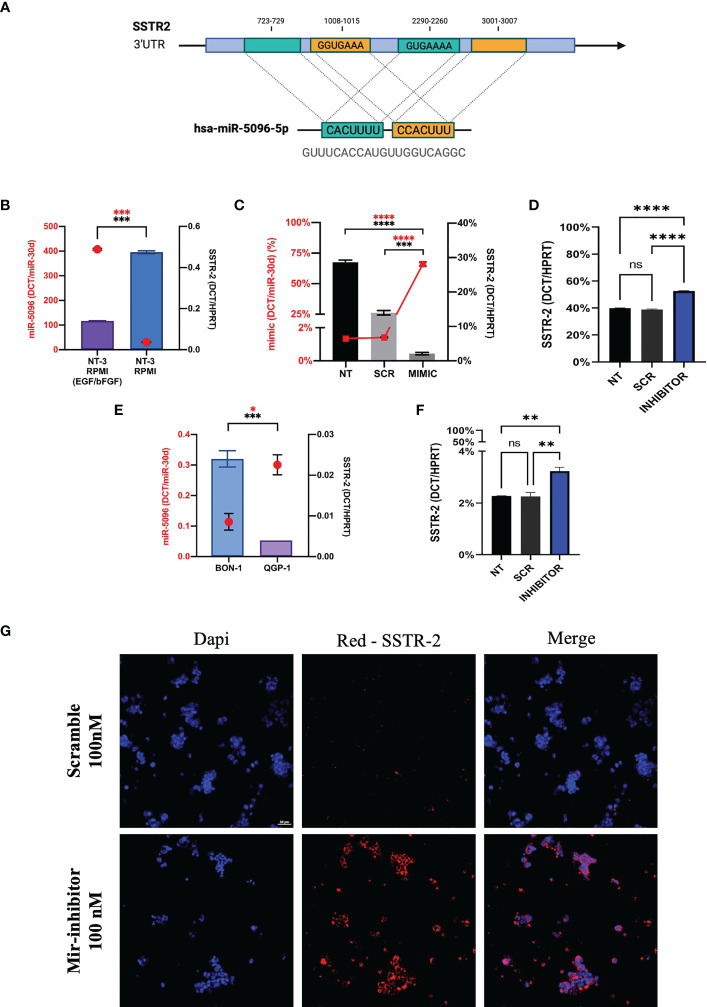
Hsa-miR-5096 overexpression down-modulates SSTR2 expression in NT-3 cell lines. **(A)** Schematic representation of hsa-mir-5096 binding sites and their relative position on SSTR2 3’UTR; **(B)** hsa-miR-5096 and SSTR2 basal expression level in low-grade NT-3 cell lines cultivated with or w/o growth factors (EGF; bFGF); **(C)** hsa-miR-5096 and SSTR2 expression in NT-3 cell lines 72h post transfection with miRCURY LNA miR-5096 mimic and scramble control; **(D)** SSTR2 expression in NT-3 cell lines, cultivated with growth factors, 72h post transfection with miRCURY LNA miR-5096 inhibitor and scramble control; **(E)** hsa-miR-5096 and SSTR2 basal expression level in high-grade BON-1 and QGP-1 cell line; **(F)** SSTR2 expression in QGP-1 cell lines, 72h post transfection with miRCURY LNA miR-5096 inhibitor and scramble control; **(G)** representative immunofluorescence staining of SSTR2 in QGP-1 cells treated with miRCURY LNA miR-5096 inhibitor and scr. *p-value<0.05; **p-value<0.01; ***p-value<0,001; ****p-value<0.0001. ns stands for non-significant.

In order to investigate the mechanism of action of hsa-miR-5096 on SSTR2 expression, we performed bioinformatic analysis with the following web-based softwares, TargetMiner, TargetScanVert and miRDB. This analysis revealed that the 3’-UTR of SSTR2 (NCBI Gene ID: 6752; GenBank Accession : NM_001050.03) harbors 4 potential binding sites for hsa-miR-5096 (miRbase Accession: MIMAT0020603; Sequence: GUUUCACCAUGUUGGUCAGGC). In particular, two different sequences (GUGAAAA; GGUGAAA) are distributed on 4 sites at the 3’-UTR of the gene (723-729; 3001-3007 and 1008-1015; 2290-2260, respectively) and are predicted to be recognized by the CACUUU and CCACUUU sequences of hsa-miR-5096. The presence of the binding sites supported a possible regulation of expression *via* direct RNA interference in PanNET tumor cells (**Figure 7A**).

In order to test this hypothesis, we performed *in vitro* experiments on the insulinoma NT-3 cell line, a newly established preclinical model of well differentiated low-grade PanNET (35). Importantly, the neuroendocrine phenotype and morphology as well as the proliferative rate and the expression of different SSTR isoforms in NT-3 cells can be modulated by the presence of growth factors (bFGF/EGF) in culture (35). SSTR2 and hsa-miR-5096 basal expression levels are inversely correlated in NT-3 cells, cultivated both with and without (w/o) growth-factors (GFs) (**Figure 7B**). In particular, SSTR2 expression was significantly enhanced (p-value<0.005) in NT-3 cells cultivated in standard RPMI (w/o GFs), characterized by low proliferation rate (10.9 +/- 0.7 days) and low ki-67 percentage (20%) (35). Conversely, hsa-miR-5096 resulted to be significantly downregulated (p-value< 0.005) in these conditions, confirming its negative correlation with SSTR2 expression and in agreement with our mechanistic model. Furthermore, NT-3 cells were ectopically treated with hsa-miR-5096-mimic while cultivated without bFGF and EGF (SSTR2^high^/hsa-miR-5096^low^ endogenous expression). As expected, the ectopic delivery of hsa-miR-5096 *via* miRCURY LNA transfection resulted in a significant intracellular increase of hsa-miR-5096/mimic, compared to not-transfected and scramble controls, confirming transfection effectiveness (p-value<0.0001; **Figure 7C**). Treatment of NT-3 cells with hsa-miR-5096 mimic for 72 hr decreased SSTR2 mRNA level of 51% as compared to scramble-treated cells (p-value<0.005; **Figure 7C**). Conversely, NT-3 cells treated with hsa-miR-5096-inhibitor while cultivated with growth-factors (SSTR2^low^/hsa-miR-5096^high^ endogenous expression) showed a significant increase in SSTR2 transcript quantity (+42%, p<0.005; **Figure 7D**). The magnitude of these modulations is in line with literature, indeed the high amount of intracellular hsa-miR-5096 mimic detected by RT/qPCR, corresponds to a modest modulation of the targets, due to the nonfunctional/activated portion of spiked mimic (37). To further substantiate the function of hsa-miR-5096 as putative post-transcriptional modulator of SSTR2 expression, its basal expression level was investigated also in preclinical models of high-grade PanNET: QGP-1 and BON-1 cells, characterized by high proliferation rate and high ki-67 percentage (about 80%). QGP-1 and BON-1 displayed significantly different amounts of SSTR2, inversely associated with significantly different hsa-miR-5096 amounts (p<0.001 and p<0.01, respectively; **Figure 7E**). Given their high amount of hsa-miR-5096, associated with low SSTR2 expression, QGP-1 cells were chosen as model to revert SSTR2 expression in high grade PanNET cells by hsa-miR-5096-inhibitor treatment. Importantly, QGP-1 treated cells showed a 39% significant increase of SSTR2 transcripts (p<0,01; **Figure 7F**) mirrored by SSTR-2 upregulation at the protein level as shown by immunofluorescence staining (**Figure 7G**). Altogether these results suggest that the delivery of specific small non-coding molecules hindering hsa-miR-5096 activity into PanNET cells can translate into *SSTR2* transcripts increased stability and higher SSTR2 amount on the cell membrane.

## Discussion

This study focuses on advanced, metastatic, and inoperable well-differentiated PanNETs, often routed to PRRT, targeting SSTRs with radiolabelled somatostatin analogues (SSAs). Nonetheless, while ^68^Ga-DOTATOC PET/CT SUV_max_ functional imaging is used to drive eligibility to PRRT and to predict its efficacy, the heterogeneous expression of SSTR2 in PanNETs affects PRRT sensitivity and accuracy (14). Indeed, despite ^68^Ga-DOTATOC PET/CT SUV_max_ helps to stratify PanNET patients, about 60% of patients do not respond to SSTR-based PRRT. Of note, PanNETs often display an increased glucose metabolism when compared to NETs from other sites of origin. Indeed. SINETs are reported to be low-metabolism neoplasms in which ^18^F-FDG-PET/CT showed lower prognostic power. On the other hand, aggressive behavior that correlates with ^18^F-FDG-PET/CT positivity and with poor PFS when treated with PRRT, suggesting a key role of glucose metabolism in the development of a PRRT refractory tumor phenotype (1, 24–28). Interestingly, we showed that specific miRNAs significantly upregulated in the blood in PanNET patients, but not in SINET ones and this might mirror an intrinsic feature of small-intestine tumors in which glucose metabolism and 18F-FDG PET/CT may not play such pivotal prognostic role in tumor aggressiveness, differently from tumors with pancreatic neuroendocrine origin. In this context our study may indicate that molecular signaling, metabolism and behavior of NETs, as well as the prognostic power of 18F-FDG PET/CT, may vary according to the different site of origin. For those reasons GEP-NETs shouldn’t be considered together as a unique entity but rather separated neoplasms with individual prognosis and management. Indeed, this study revealed that SI-NETs subset expressed higher levels of circulating hsa-miR-5096 when compared to PanNET patients and/or healthy donors (data not shown), nevertheless hsa-miR-5096 were not able to correlate with increased tumor metabolism or 18F-FDG PET/CT positivity. Hence, both functional imaging with ^18^F-FDG PET/CT and ^68^Ga-DOTATOC PET/CT have shown to be prognostic and predictive, but with some limitations, such as the difficulty of quantifying the uptake and a lack of standardization for the uptake from multiple lesions. In this framework, it is still of clinical relevance to *i)* better understand the biology of these tumors, investigating molecular mechanisms leading to a PRRT refractory phenotype; *ii)* improve prognostic and predictive algorithms and provide better stratification of PanNETs undergoing PRRT. The multinational, multidisciplinary Delphi consensus encouraged multi-analyte measurements usage to provide more accurate information on the proliferative, metabolic, and metastatic features of NETs (4). In this context, the combination of *in vivo* spatial and functional imaging of the tumor with measurable circulating transcripts (mRNAs and ncRNAs) should be preferred and could represent a key strategy for real-time disease monitoring and prognostication in the near future (9). Currently, the only approved *in vitro diagnostic* (IVD) tool for NETs is the NETest. Recently, NETest was combined with grading and used to generate a PRRT predictive quotient (PPQ) for NETs. However, the NETest does not consider neither the contribution of tumor metabolism nor a direct correlation with ^18^F-FDG-PET/CT status (38–42). Our results suggest a potential role of hsa-mir-5096 alone and combined into predictor P2 as oligo-analyte for ^18^F-FDG/PET positivity in PanNET patients (**Supplementary Tables S2, S3**). In this context, *“Predictors”* may be useful to build a multi-analyte assay, given the possibility to mathematically combine the prognostic power of two or more miRNAs within a single blood withdrawal. However, we did not observe any significant differences between AUCs values, of single hsa-miR-5096 performances as compared to “*Predictors*” in terms of 6-mPFS and 12-mOS; while P2 can be used to best predict ^18^F-FDG PET/CT endpoint only when multi-analyte measurements are preferred (see **Supplementary File S3**, **Supplementary Figures S2-S4**, **Tables S2, S3**). In addition, ROC curve and KM analysis revealed that hsa-miR-5096 can perform as an accurate and independent predictor of PFS in PanNET patients treated with ^177^Lu-DOTATATE PRRT with 90% accuracy. In our retrospective study, our assay exhibits a metric comparable with the NETest that currently differentiate PanNET stable disease from progressive disease with 85% accuracy, considering the expression of 51-transcripts through a dedicated algorithmic analysis provided at the moment by a single lab in the world (38). Of clinical relevance, the combination of ^18^F-FDG PET/CT positivity with a value of hsa-miR-5096>70 identifies a novel prognostic category characterized by the poorest PFS after ^177^Lu-DOTATATE PRRT. Cut-off of 70 shows similar performance to LIU/Yuden *standard computed* cut-off but it avoids false positives at ^18^F-FDG PET/CT, preventing overtreatment of negative patients (hsa-miR-5096 <70). Of clinical relevance, the same cut-off of 70 well performs for different endpoints, thus increasing the clinical utility of this marker and facilitating the interpretation of results. Despite further external validation on independent cohorts of PanNET patients that is required, hsa-mir-5096 cam be considered as a companion biomarker of ^18^F-FDG PET/CT to improve PanNETs stratification and predictivity of PRRT efficacy. In this context, hsa-miR-5096 assessment constitutes a low complexity, minimally invasive assay that requires basic and already broadly diffused equipment for being assessed in hospitals. Hsa-miR-5096 could be also considered as a potential candidate “Type II” biomarker (38). The described prognostic effect of hsa-mir-5096 is independent from all standard clinical parameters taken into consideration, except from grading which is expected given the different biology of positive and negative ^18^F-FDG PET/CT PanNETs. Interestingly, circulating hsa-miR-5096 showed a mild inverse correlation with ^68^Ga-DOTATOC PET/CT SUV_max_, and this negative correlation of 68Ga-DOTATOC PET/CT SUV_max_ associated with ^18^F-FDG-PET/CT positivity in patients displaying low and high levels of hsa-miR-5096 (cut-off:70; **Figure 6B**), identifies a subgroup of ^18^F-FDG-PET positive patients with higher density of SSTR2 receptors, similar to ^18^F-FDG-PET negative ones, this might help to address PRRT schedule preventing overtreatments and supporting the role of hsa-miR-5096 as a companion biomarker for patients’ stratification. These observations were conducted considering a relatively limited sample size; thus we overcame this limitation, further confirming the inverse correlation of SSTR2 and hsa-miR-5096 also at the single cell level on PanNET tissue specimens. In this context, we set up the miR-Protein *in situ* protocol to detect on the same tissue section both markers, using a semi-automated and robust procedure which also allowed us to save valuable patient’s material. Of note, the novel miR-Protein detection and dedicated AND-Tool software of analysis provide the simultaneous detection of miRNAs and proteins, followed by standardized, operator independent measurements, turning qualitative *in situ* revelation into a quantitative analysis. Specifically, our novel staining workflow allows the automatization and avoids antigen degradation which typically occurs when immunohistochemistry is performed prior to ISH. In addition, the usage of DAB-brown staining, in contrast to typical blue used for ISH labeling, was crucial to ensure miRNA staining stability and to discriminate DAB-brown positive from negative nuclei (counterstained with hematoxylin) allowing AND-Tool software-based analysis. We believe our results sustain hsa-miR-5096 direct involvement in SSTR2 turnover into PanNET cells. Indeed, hsa-miR-5096 ectopic overexpression in PanNET insulinoma NT-3 cells led to a significant decrease of SSTR2 transcripts, while hsa-miR-5096 inhibition significantly boosted SSTR2 expression both in QGP-1 and NT-3 substantiating direct targeting and regulation in PanNETs characterized by SSTR2^low^/hsa-miR-5096^high^ phenotype. Notably, NT-3 cells treated with growth factors are characterized by increased ki-67%, hsa-miR-5096 induction and decreased SSTR2 level, consistent with a more aggressive phenotype and with data observed in patients. From this perspective hsa-miR-5096 seems to contribute to a metabolic switch leading to lineage differentiation in PanNET cells. Of note, hsa-miR-5096 has been involved in glioblastoma biology and reported to be overexpressed in breast cancer and binds with high affinity about 725 target genes (42–44).

Several limitations of our study must be acknowledged for correct interpretation of the results. Owing to the indolent nature of GEP-NETs and their rarity, especially of PanNETs and given the enrolment rate of LUX and LUNET clinical trials we are aware that the sample size of our screening and validation cohorts are relatively limited and include different tumor types, making our case series not-equally distributed in terms of primary site of origin. Specifically, the Univariate Cox regression model should be considered explorative, to provide indication of the biomarker independent predictivity and further validation on enlarged cohorts is desirable. In addition, the retrospective design, the enrollment rate, may have affected the population distribution of the validation cohort in terms of sex, age, or grade. To overcome this limitation, we refute age contribution to miRNAs signature deregulation in plasma of PanNET patients (**Supplementary File**, **Supplementary Figure S1**) and we introduced clear and strict inclusion criteria, performed a comprehensive evaluation of clinical and tissue-based parameters, and performed blinded radiological review of all baseline and restaging images in order to maximize the information value and quality of data derived from this investigation. In order to provide adequate statistical power, robustness and translatability for clinical management we encourage further validation on external, enlarged and independent prospective cohorts. The monocentric nature of the present study and the rarity of the disease may also represent limitations since patients from different parts of the country performed follow-up elsewhere and resulted lost to follow-up, preventing the collection of an adequate and homogeneous follow-up case series. Here, we assessed hsa-miR-5096 circulating levels at baseline, focusing on its performance as prognostic biomarker for patient stratification prior to PRRT treatment, alone or in combination with hsa-miR-let7i (P2, if multianalyte assessment is preferred) and on its functional role in PanNET biology and SSTR2 modulation, providing the proof of concept of potential therapeutic compounds. In this context, we performed *in vitro* experiments on NT-3 cells that are the most representative, and validated cell model for low grade PanNET studies. Indeed, NT-3 cells express SSTR1, 2, 3 and 5 (35) isoforms and our analysis focused on *SSTR2* which contains multiple target sequences for hsa-miR-5096 in its 3’-UTR and because of its prevalence and clinical relevance in PanNETs, as target of PRRT. Interestingly, we have found that also the 3’-UTR of SSTR3 harbors a single hsa-miR-5096 predicted binding site (TargetScan_Vert source). SSTR3 may also determine a modulation of SSTR turnover and signaling in NET disease. In addition, TargetScan_Vert based analysis also reported 5 binding sites on the 3’UTR of the Somatostatin gene (SST). SST is a small peptide that binds SSTRs exerting inhibitory effects on neuroendocrine cells, including cell growth and hormone release inhibition. Indeed, SST analogs are employed to treat neuroendocrine disease and novel epigenetic regulators of SST signaling or SSA–mTOR inhibitors have been recently proposed as combination therapy for tumor control. Alongside classical SSA treatment regimens, future advanced therapies are expected to improve the management of NET patients. Virtually, hsa-mir-5096-5p can interfere with both receptors and ligands downmodulating the whole signaling, thus blocking hsa-mir-5096-5p could result in decreased tumor growth and increased sensitivity to PRRT. Given this, even if it was beyond the scope of the present study, we believe both SST and SSTR3 warrant further exploration in relation to the here defined miRNA. In addition, the observation on high grade PanNET BON-1 and QGP-1 cell lines further supported the existence of a hsa-miR-5096-SSTR2 axis and the hsa-miR-5096 mediated interference on SSTR2 transcripts. Accordingly, hsa-miR-5096 inhibitor was more effective on QGP-1 cells in triggering a significant SSTR2 upregulation since QGP-1 display higher levels of hsa-miR-5096 and lower SSTR-2 amounts compared to BON-1 cells. Our results lay the conceptual basis for a novel therapeutic for PanNET management, in order to sensitize tumor cells to PRRT via the delivery of specific hsa-miR-5096 inhibitory molecules.

## Conclusions

To conclude our study has led to a candidate prognostic and low-complexity miRNA signature easily retrievable in plasma of PanNET patients. The potential clinical utility of hsa-miR-5096 alone or in combination with hsa-let-7i-3p relies on its prognostic power in predicting metabolic aggressiveness which will help in PanNET stratification treated with PRRT. Moreover, our findings suggest that PanNET tumor cells can produce higher amounts of resident hsa-miR-5096 downmodulating SSTR2 with autocrine mechanism or shedding increased amounts in biofluids *via* exosomes, resulting in SSTR2 expression in recipient cells *via* a paracrine mechanism. This mechanism may contribute to tumor heterogeneity and to the development of a refractory phenotype and/or relapse to PRRT. Importantly, our findings suggest that a therapeutic approach aimed to interfere with hsa-miR-5096 activity, inhibiting its targeting of SSTR2 3’-UTR sequences, would enhance SSTRs expression and sensitize tumor cells to PRRT or other SSTR-targeted therapies. Current efforts are heading in this direction to provide PanNET patients with additional and more efficacious therapeutic options.

## Data availability statement

The data presented in the study are deposited in the European Genome-phenome Archive (EGA) repository, accession number EGAD00001010840.

## Ethics statement

The studies involving human participants were reviewed and approved by Centro di Coordinamento Studi IRST IRCCS Unità di Biostatistica e Sperimentazioni Cliniche (CEROM), approval no. 6711/5.1/2016. The patients/participants provided their written informed consent to participate in this study.

## Author contributions

MB, MM, and GP conceived and developed the idea of the study. MB performed experiments. RC and MA performed NGS analysis. MB, MiT, and FF analyzed the data. SS and IG provided the surgical specimens. MB and SR set up the protocols and performed miRNA-ISH/IHC on tumor tissue specimens. MaT acquired and scanned the images and FP designed and provided the AND-Tool software. JS kindly provided NT-3, QGP-1 and BON-1 cell lines and contributed with insightful observations, discussion of the data and technical support. MB drafted the manuscript. MB, MaT, FP, FN and MM were responsible for data interpretation and revised the manuscript. All authors read and approved the final version of the manuscript for submission.

## References

[1] Andreasi V Partelli S Muffatti F Manzoni MF Capurso G Falconi M. Update on gastroenteropancreatic neuroendocrine tumors. Dig Liver Dis (2021) 53:171–82. doi: 10.1016/j.dld.2020.08.031 32912771

[2] Dasari A Shen C Halperin D Zhao B Zhou S Xu Y. Trends in the incidence, prevalence, and survival outcomes in patients with neuroendocrine tumors in the united states. JAMA Oncol (2017) 3:1335–42. doi: 10.1001/jamaoncol.2017.0589 PMC5824320 28448665

[3] Young K Starling N Sadanandam A. The molecular biology of pancreatic neuroendocrine neoplasms: challenges and translational opportunities. Semin Cancer Biol (2020) 61:132–8. doi: 10.1016/j.semcancer.2019.09.024 31577961

[4] Oberg K Krenning E Sundin A Bodei L Kidd M Tesselaar M. A Delphic consensus assessment: imaging and biomarkers in gastroenteropancreatic neuroendocrine tumor disease management. Endocr Connect (2016) 5:174–87. doi: 10.1530/EC-16-0043 PMC5045519 27582247

[5] Howe JR Merchant NB Conrad C Keutgen XM Hallet J Drebin JA. The north American neuroendocrine tumor society consensus paper on the surgical management of pancreatic neuroendocrine tumors. Pancreas (2020) 49:1–33. doi: 10.1097/MPA.0000000000001454 31856076 PMC7029300

[6] Mafficini A Scarpa A. Genomic landscape of pancreatic neuroendocrine tumours: the international cancer genome consortium. J Endocrinol (2018) 236:R161–7. doi: 10.1530/JOE-17-0560 PMC5811627 29321190

[7] Scarpa A Chang DK Nones K Corbo V Patch AM Bailey P. Corrigendum: whole-genome landscape of pancreatic neuroendocrine tumours. Nature (2017) 550:548. doi: 10.1038/nature24026 28953865

[8] Partelli S Bartsch DK Capdevila J Chen J Knigge U Niederle B. ENETS consensus guidelines for standard of care in neuroendocrine tumours: surgery for small intestinal and pancreatic neuroendocrine tumours. Neuroendocrinology (2017) 105:255–65. doi: 10.1159/000464292 28237989

[9] Modlin IM Moss SF Chung DC Jensen RT Snyderwine E. Priorities for improving the management of gastroenteropancreatic neuroendocrine tumors. J Natl Cancer Inst (2008) 100:1282–9. doi: 10.1093/jnci/djn275 PMC2538549 18780869

[10] Ahmed M. Gastrointestinal neuroendocrine tumors in 2020. World J Gastrointest Oncol (2020) 12:791–807. 32879660 10.4251/wjgo.v12.i8.791 PMC7443843

[11] Nagtegaal ID Odze RD Klimstra D Paradis V Rugge M Schirmacher P. WHO classification of tumours editorial board, 2020. the 2019 WHO classification of tumours of the digestive system. Histopathology (2020) 76:182–8. doi: 10.1111/his.13975 PMC7003895 31433515

[12] Inzani F Petrone G Rindi G. The new world health organization classification for pancreatic neuroendocrine neoplasia. Endocrinol Metab Clin North Am (2018) 47:463–70. doi: 10.1016/j.ecl.2018.04.008 30098710

[13] Choe J Kim KW Kim HJ Kim DW Kim KP Hong SM. What is new in the 2017 world health organization classification and 8th American joint committee on cancer staging system for pancreatic neuroendocrine neoplasms? Korean J Radiol (2019) 20:5–17. doi: 10.3348/kjr.2018.0040 30627018 PMC6315069

[14] Guenter R Aweda T Carmona Matos DM Jang S Whitt J Cheng YQ. Overexpression of somatostatin receptor type 2 in neuroendocrine tumors for improved Ga68-DOTATATE imaging and treatment. Surg (United States) (2020) 167(1):189–96. doi: 10.1016/j.surg.2019.05.092 PMC8422841 31629542

[15] Bodei L Ambrosini V Herrmann K Modlin I. Current concepts in (68)Ga-DOTATATE imaging of neuroendocrine neoplasms: interpretation, biodistribution, dosimetry, and molecular strategies. J Nucl Med (2017) 58:1718–26. doi: 10.2967/jnumed.116.186361 28818983

[16] Oberg KE Reubi JC Kwekkeboom DJ Krenning EP. Role of somatostatins in gastroenteropancreatic neuroendocrine tumor development and therapy. Gastroenterology 139 (2010) 742-53:753.e1. doi: 10.1053/j.gastro.2010.07.002 20637207

[17] Lepage C Dahan L Bouarioua N Toumpanakis C Legoux JL Le Malicot K. Evaluating lanreotide as maintenance therapy after first-line treatment in patients with non-resectable duodeno-pancreatic neuroendocrine tumours. Dig Liver Dis (2017) 49:568–71. doi: 10.1016/j.dld.2017.02.004 28292641

[18] Caplin ME Pavel M Cwikla JB Phan AT Raderer M Sedláčková M. Lanreotide in metastatic enteropancreatic neuroendocrine tumors. N Engl J Med (2014) 371:224–33. doi: 10.1056/NEJMoa1316158 25014687

[19] Bodei L Kidd MS Singh A Zwan W Severi S Drozdov IA. PRRT neuroendocrine tumor response monitored using circulating transcript analysis: the NETest. Eur J Nucl Med Mol Imaging (2020) 47:895–906. doi: 10.1007/s00259-019-04601-3 31838581 PMC7515632

[20] Bodei L Kidd MS Singh A van der Zwan WA Severi S Drozdov IA. PRRT genomic signature in blood for prediction of (177)Lu-octreotate efficacy. Eur J Nucl Med Mol Imaging (2018) 45:1155–69. doi: 10.1007/s00259-018-3967-6 PMC6716527 29484451

[21] Baum RP Kulkarni HR Singh A Kaemmerer D Mueller D Prasad V. Results and adverse events of personalized peptide receptor radionuclide therapy with (90)Yttrium and (177)Lutetium in 1048 patients with neuroendocrine neoplasms. Oncotarget (2018) 9:16932–50. doi: 10.18632/oncotarget.24524 PMC5908296 29682195

[22] Kwekkeboom DJ Kam BL van Essen M Teunissen JJ van Eijck CH Valkema R. Somatostatin-receptor-based imaging and therapy of gastroenteropancreatic neuroendocrine tumors. Endocr Relat Cancer (2010) 17:R53–73. doi: 10.1677/ERC-09-0078 19995807

[23] Bodei L Schoder H Baum RP Herrmann K Strosberg J Caplin M. Molecular profiling of neuroendocrine tumours to predict response and toxicity to peptide receptor radionuclide therapy. Lancet Oncol (2020) 21:e431–43. doi: 10.1016/S1470-2045(20)30323-5 PMC8385643 32888472

[24] Das S Al-Toubah T El-Haddad G Strosberg J. (177)Lu-DOTATATE for the treatment of gastroenteropancreatic neuroendocrine tumors. Expert Rev. Gastroenterol. Hepatol (2019) 13:1023–31. doi: 10.1080/17474124.2019.1685381 PMC7227421 31652074

[25] Sundin A Arnold R Baudin E Cwikla JB Eriksson B Fanti S. ENETS consensus guidelines for the standards of care in neuroendocrine tumors: radiological, nuclear medicine & hybrid imaging. Neuroendocrinology (2017) 105:212–44. doi: 10.1159/000471879 28355596

[26] Severi S Nanni O Bodei L Sansovini M Ianniello A Nicoletti S. Role of 18FDG PET/CT in patients treated with 177Lu-DOTATATE for advanced differentiated neuroendocrine tumours. Eur J Nucl Med Mol Imaging (2013) 40:881–8. doi: 10.1007/s00259-013-2369-z 23443937

[27] Larouche V Akirov A Alshehri S Ezzat S. Management of small bowel neuroendocrine tumors. Cancers (Basel) (2019) 11(9):1395. doi: 10.3390/cancers11091395 31540509 PMC6770692

[28] Bocchini M Nicolini F Severi S Bongiovanni A Ibrahim T Simonetti G. Biomarkers for pancreatic neuroendocrine neoplasms (PanNENs) management-an updated review. Front Oncol (2020) 10:831. doi: 10.3389/fonc.2020.00831 32537434 PMC7267066

[29] Nik Mohamed Kamal NNSB Shahidan WNS. Non-exosomal and exosomal circulatory MicroRNAs: which are more valid as biomarkers? Front Pharmacol (2020) 10:1500. doi: 10.3389/fphar.2019.01500 32038230 PMC6984169

[30] Beccuti M Cordero F Arigoni M Panero R Amparore EG Donatelli S. SeqBox: RNAseq/ChIPseq reproducible analysis on a consumer game computer. Bioinformatics (2018) 34:871–2. doi: 10.1093/bioinformatics/btx674 PMC6030956 29069297

[31] Kulkarni N Alessandri L Panero R Arigoni M Olivero M Ferrero G. Reproducible bioinformatics project: a community for reproducible bioinformatics analysis pipelines. BMC Bioinf (2018) 19(Suppl 10):349. doi: 10.1186/s12859-018-2296-x PMC6191970 30367595

[32] David M Dzamba M Lister D Ilie L Brudno M. SHRiMP2: sensitive yet practical SHort read mapping. Bioinformatics (2011) 27:1011–2. doi: 10.1093/bioinformatics/btr046 21278192

[33] Love MI Huber W Anders S. Moderated estimation of fold change and dispersion for RNA-seq data with DESeq2. Genome Biol (2014) 15:550–014-0550-8. doi: 10.1186/s13059-014-0550-8 25516281 PMC4302049

[34] Landini G Martinelli G Piccinini F. Colour deconvolution: stain unmixing in histological imaging. Bioinformatics (2021) 37:1485–7. doi: 10.1093/bioinformatics/btaa847 32997742

[35] Benten D Behrang Y Unrau L Weissmann V Wolters-Eisfeld G Burdak-Rothkamm S. Establishment of the first well-differentiated human pancreatic neuroendocrine tumor model. Mol Cancer Res (2018) 16:496–507. 29330294 10.1158/1541-7786.MCR-17-0163

[36] DeLong ER DeLong DM Clarke-Pearson DL. Comparing the areas under two or more correlated receiver operating characteristic curves: a nonparametric approach. Biometrics (1988) 44:837–45. doi: 10.2307/2531595 3203132

[37] Thomson DW Bracken CP Szubert JM Goodall GJ. On measuring miRNAs after transient transfection of mimics or antisense inhibitors. PloS One (2013) 8:e55214. doi: 10.1371/journal.pone.0055214 23358900 PMC3554668

[38] Oberg K Califano A Strosberg JR Ma S Pape U Bodei L. A meta-analysis of the accuracy of a neuroendocrine tumor mRNA genomic biomarker (NETest) in blood. Ann Oncol (2020) 31:202–12. doi: 10.1016/j.annonc.2019.11.003 31959337

[39] Frank R Hargreaves R. Clinical biomarkers in drug discovery and development. Nat Rev Drug Discovery (2003) 2:566–80. doi: 10.1038/nrd1130 12838269

[40] Bodei L Kidd M Modlin IM Severi S Drozdov I Nicolini S. Measurement of circulating transcripts and gene cluster analysis predicts and defines therapeutic efficacy of peptide receptor radionuclide therapy (PRRT) in neuroendocrine tumors. Eur J Nucl Med Mol Imaging (2016) 43:839–51. doi: 10.1007/s00259-015-3250-z 26596723

[41] Malczewska A Bodei L Kidd M Modlin IM. Blood mRNA measurement (NETest) for neuroendocrine tumor diagnosis of image-negative liver metastatic disease. J Clin Endocrinol Metab (2019) 104:867–72. doi: 10.1210/jc.2018-01804 30358858

[42] Thuringer D Boucher J Jego G Pernet N Cronier L Hammann A. Transfer of functional microRNAs between glioblastoma and microvascular endothelial cells through gap junctions. Oncotarget (2016) 7:73925–34. doi: 10.18632/oncotarget.12136 PMC5342024 27661112

[43] Buruiana A Florian SI Florian AI Timiș TL Mihu CM Miclăuș M. The roles of miRNA in glioblastoma tumor cell communication: diplomatic and aggressive negotiations. Int J Mol Sci (2020) 21(6):1950. doi: 10.3390/ijms21061950 32178454 PMC7139390

[44] Ivashchenko A Berillo O Pyrkova A Niyazova R Atambayeva S. The properties of binding sites of miR-619-5p, miR-5095, miR-5096, and miR-5585-3p in the mRNAs of human genes. BioMed Res Int (2014) 2014:720715. doi: 10.1155/2014/720715 25162022 PMC4137733

