# Supplementary material for: Circulating hsa-miR-5096 predicts 18F-FDG PET/CT positivity and modulates somatostatin receptor 2 expression: a novel miR-based assay for pancreatic neuroendocrine tumors
Source: Front Oncol. 2023 May 23;13:1136331. doi: 10.3389/fonc.2023.1136331 (PMC10242108; doi:10.3389/fonc.2023.1136331)
Supplement: Supplementary file 1 [file DataSheet_1.docx]

**Supplementary Table S1 Demographic and clinical pathological al features according to ^18^FDG/PET outcome: PAN-NEN case series (n= 38)**

| **Variable** | **^18^FDG/PET Positive**  **(%)**  **N = 25** | **^18^FDG/PET Negative**  **(%)**  **N = 13** | **Healthy**  **donors**  **(%)**  **N = 17** | **Overall** | **p-value** |
| --- | --- | --- | --- | --- | --- |
| **Age at ^18^PET-FDG** |  |  |  |  |  |
| **Median (range)** | 52 (24-78) | 61 (44-79) | 42 (30-79) | 54 (30-79) | 0.046 |
|  |  |  |  |  |  |
| **Gender** |  |  |  |  |  |
| **Male** | 16 (64.0) | 11 (84.6) | 11 (64.7) | 38 (69.1) | 0.038 |
| **Female** | 9 (36.0) | 2 (15.4) | 6 (35.3) | 17 (30.9) |  |
|  |  |  |  |  |  |
| **Ki67** |  |  |  |  |  |
| **Median (range)** | 12 (1-50) | 2 (1-15) |  | 8 (1-50) | 0.003 |
|  |  |  |  |  |  |
| **Grading (WHO 2017)** |  |  |  |  |  |
| **1** | 4 (16.0) | 8 (61.5) |  | 12 (31.6) | 0.015 |
| **2** | 17 (68.0) | 5 (38.5) |  | 22 (57.9) |  |
| **3** | 4 (16.0) | 0 (0.0) |  | 4 (10.5) |  |
|  |  |  |  |  |  |
| **Tumor burden** |  |  |  |  |  |
| **Limited** | 6 (24.0) | 5 (38.5) |  | 11 (28.0) | 0.680 |
| **Moderate** | 12 (48.0) | 5 (38.5) |  | 17 (44.7) |  |
| **Extensive** | 7 (28.0) | 3 (23.0) |  | 10 (26.3) |  |
|  |  |  |  |  |  |
| **Number of metastatic sites** |  |  |  |  |  |
| **None** | 4 (16.0) | 1 (7.7) |  | 5 (13.2) | 0.282 |
| **One** | 9 (36.0) | 4 (30.8) |  | 13 (34.2) |  |
| **Two** | 8 (32.0) | 8 (61.5) |  | 16 (42.1) |  |
| **Three** | 4 (16.0) | 0 (0.0) |  | 4 (10.5) |  |
|  |  |  |  |  |  |
| **Liver lesions** |  |  |  |  | 0.817 |
| **None** | 5 (20.8) | 2 (15.4) |  | 7 (18.9) |  |
| **<6** | 7 (29.2) | 5 (38.5) |  | 12 (32.4) |  |
| **>=6** | 12 (50.0) | 6 (46.1) |  | 18 (48.7) |  |
| **Unknown** | 1 | 0 |  | 1 |  |
|  |  |  |  |  |  |
| **Presence of bone metastasis** | 6 (24.0) | 3 (23.1) |  | 9 (23.7) | 1.000 |
|  |  |  |  |  |  |
| **Rotterdam Index** |  |  |  |  |  |
| **3** | 4 (16.0) | 6 (60.0) |  | 10 (28.6) | 0.016 |
| **4** | 21 (84.0) | 4 (40.0) |  | 25 (71.4) |  |
|  |  |  |  |  |  |
| **SUV PET GA** |  |  |  |  |  |
| **Median (range)** | 33 (5.8-93) | 62.5 (15.0-257) |  | 36.9 (5.8-257) | 0.077 |
| **Unknown** | 9 | 12 |  | 12 |  |

p-value from Fisher exact test for categorical variables and Wilcoxon Mann-Whitney for continuous variable

**Supplementary 1 (S1) Data registry collection and statistical analysis of PanNET (38) population distribution**

Demographic (year of birth, age to diagnosis and gender) and clinical pathological features of the disease (site of origin, ki-67%, grading, tumor burden, presence and number of bone and/or liver metastases, 68Gallium-PET SUVmax, 18F - FDG/PET/PET SUVmax ) were collected.

Chi-square test or Fisher exact test for categorical data were applied on Pan-NEN subset, while Wilcoxon Mann-Whitney test was used for continuous data. Normality of distribution was assessed through Shapiro-Wilk test. Kruskall Wallis test was performed, according to age, to assess discrepancies between the Pan-NEN subset according to 18F - FDG/PET status and healthy donors’ cohort. Dunn’s test was performed as a post-hoc comparison.

Progression free survival (PFS) and overall Survival (OS) were calculated according to the Kaplan-Meier method and 95% confidence interval was provided (95%CI) . Statistical analysis was performed to evaluate the presence of potential confounding factors on 38 Pan-NEN patients’ subset according to 18F - FDG/PET outcome (see Tab. S1 for Pan-NET for clinico-pathological features). In particular, to exclude the potential role of age as a confounder we evaluated the miR-signature expression value in Pan-NENs and healthy donors according to PanNEN population median age (cut-off: 54.5 years) (see Fig.S1 for age contribution to miR-signature and predictors expression level in plasma of Pan-NENs and Healthy Donors, according to 18F-FDG/PET).

Median age of 38 PAN-NETs subset was 54.5 years (range: 24-79 years): among PAN-NET cohort, 18F - FDG/PET positive patients (n=25) displayed 52 years (range: 24-78 years) as median age while 18F - FDG/PET negative patients showed 61 years (range: 44-79 years) as median age, with a statistical difference among the two groups (0.011). In addition, a significant difference in terms of age between PAN-NET patients and healthy donors (p<0.05), further addressing the issue of age as a confounder.

To exclude the potential role of age as confounder we evaluated the miR-signature expression value in Pan-NETs and healthy donors according to PAN-NET population median age (cut-off: 54.5 years). Of primary relevance, no significant differences were highlighted in miR-signature expression level between younger (<54.5) and elder (>54.5), excluding the possible contribute of age in determining miRNAs associated with the 18F - FDG/PET outcome (see Fig.S1 for age contribution to miR-signature and predictors expression level in plasma of Pan-NENs and Healthy Donors, according to 18F-FDG/PET). Finally, no significant differences in gender of patients and donors populations emerged.

Median PFS was 41.1(95%CI:18.5%-45.2) while median OS was not reached at the time of the analysis and 12 months OS was 94.1 (95%CI:78.5-98.5) .

**Supplementary Figure S1 Age contribution to miR-signature and predictors expression level in plasma of PAN-NENs and Healthy Donors (cut-off: 54.5), according to ^18^F-FDG/PET**

**
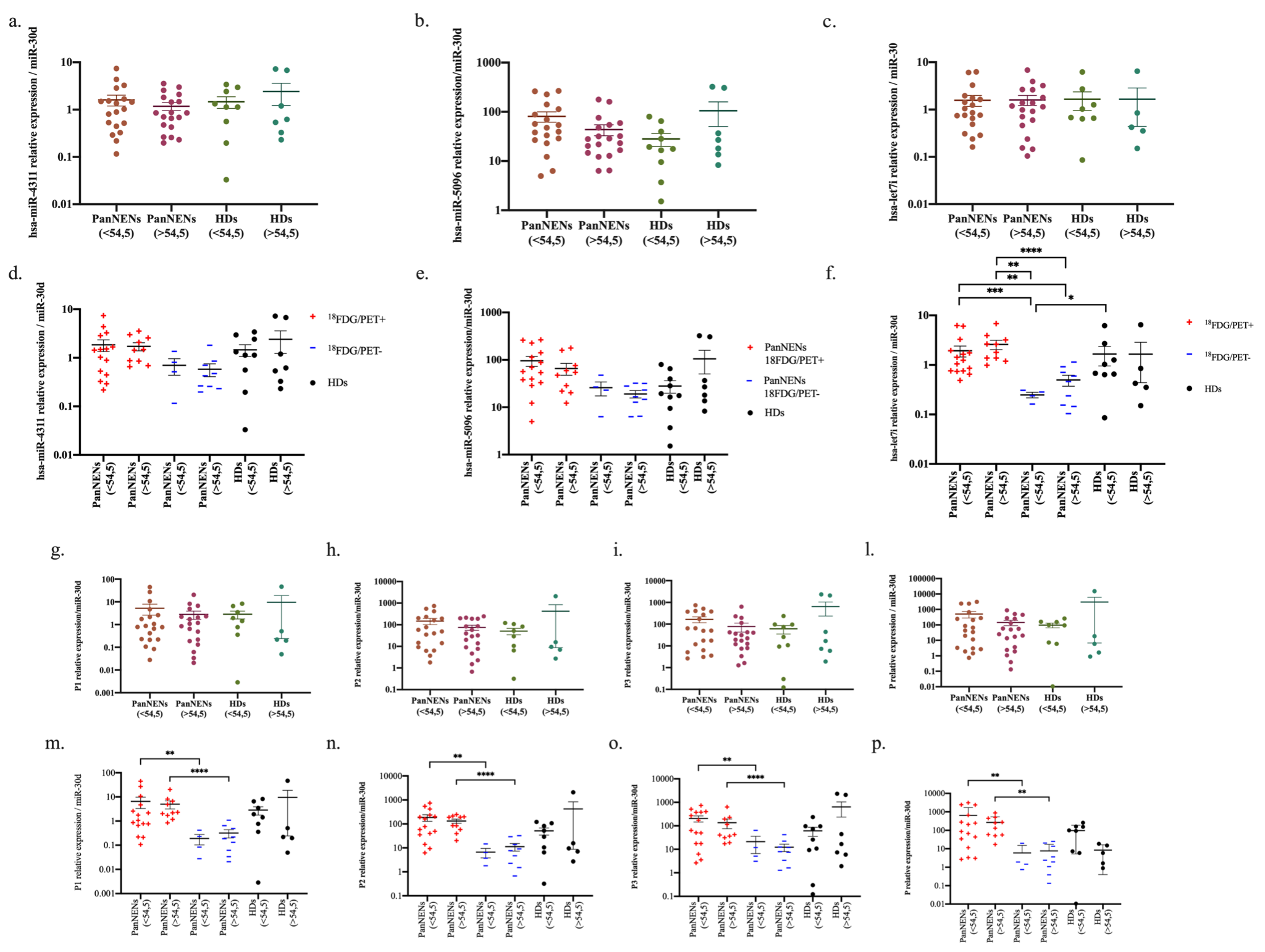
**

**Figure legend:**

Age contribution to single miR expression level in plasma of PAN-NENs and Healthy Donors (a-c): (a) hsa-miR-4311; (b) hsa-miR-5096; (c) hsa-let-7i-3p. Age contribution to single miR expression level in plasma of PAN-NENs and Healthy Donors, according to ^18^F-FDG/PET (e-g): (e) hsa-miR-4311, in ^18^F-FDG/PET positive and negative patients; (f) hsa-miR-5096, in ^18^F-FDG/PET positive and negative patients; (g) hsa-let-7i-3p,in ^18^F-FDG/PET positive and negative patients. Age contribution to predictors expression level in plasma of PAN-NENs and Healthy Donors, according to ^18^F-FDG/PET (m-p): (m) P1 (hsa-miR-4311* hsa-let-7i-3p), in ^18^F-FDG/PET positive and negative patients;.; (h) P2 (hsa-mir-5096*hsa-let-7i-3p), in ^18^F-FDG/PET positive and negative patients;.; (i) P3 (hsa-miR-4311* hsa-mir-5096), in ^18^F-FDG/PET positive and negative patients; (l) P (hsa-miR-4311*hsa-mir-5096*hsa-let-7i-3p), in ^18^F-FDG/PET positive and negative patients. Healthy Donors = HDs

**Supplementary File S2** AND-Tool user manual (PDF)

**Supplementary File S3:** Predictors were mathematically built multiplying the fold enrichment value obtained for the single miRNAs in different combinations. In order to obtain the fold enrichment for the single miRNAs, each miRNA expression level was normalized on the level of the reference hsa-miR-30d through a standard 2-∆CT method.

We calculated 3 binary predictors by combining, hsa-miR-4311*hsa-let-7i-3p (P1), hsa-miR-5096*hsa-let-7i-3p (P2), hsa-miR-4311*hsa-miR-5096 (P3) fold changes, and 1 triple predictor which combines all 3 fold changes, hsa-miR-4311*hsa-miR-5096*hsa-let-7i-3p (P), in a single value.

**Supplementary Figure S2 qPCR validation of combined predictors (P1, P2, P3 and P) to define multi-analyte biomarker that correlates with ^18^ FDG/PET**

**
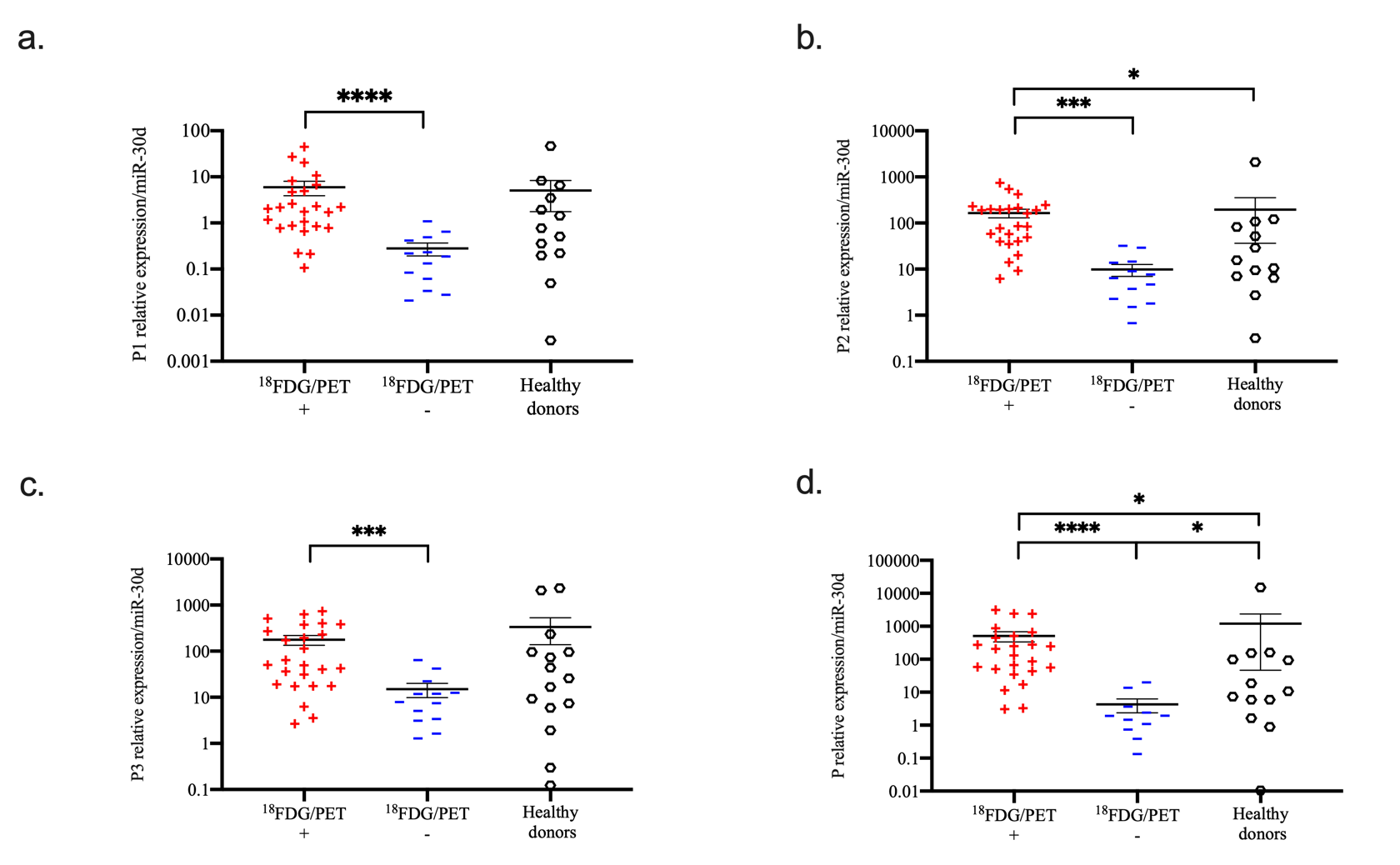
**

**Figure legend (S2) :** (a-d) Deregulated predictor between 18F-FDG/PET positive and negative PanNETs: (a) P1 (hsa-miR-4311* hsa-let-7i-3p), (b) P2 (hsa-mir-5096*hsa-let-7i-3p), (c) P3 (hsa-miR-4311* hsa-mir-5096), (d) P (hsa-miR-4311*hsa-mir-5096*hsa-let-7i-3p). Hsa-miR-30d was selected from NGS profiling as an endogenous control. Results are presented as mean ± SD (*p-value<0.05; ****p-value<0.00005). Each graph was represented in Log10 scale and Wilcoxon and Mann-Whitney test, chi-square tests were applied respectively for continuous data and categorical data. For comparison between three groups Kruskal-Wallis test was used and Dunn test was used for post-hoc comparisons.

**Supplementary Table S2** Single miRNAs and combined predictors (P1, P2, P3 and, P) expression values in P-NET ^18^FDG/PET positive and negative patients and Healthy Donors (HDs)

| **miR-** | **^18^FDG/PET Positive**  **N = 25** | **^18^FDG/PET Negative**  **N = 13** | **Healthy**  **donors**  **N = 17** | **Overall**  **N = 55** | **p-value** | **p-value (neg. *vs* HDs)** | **p-value (pos. *vs* HDs)** | **p-value (pos. *vs* neg)** |
| --- | --- | --- | --- | --- | --- | --- | --- | --- |
|  |  |  |  |  |  |  |  |  |
| **Has-miR-4311** |  |  |  |  |  |  |  |  |
| Median (range) | 1.44  (0.21-7.38) | 0.47  (0.11-1.81) | 1.13  (0.03-7.21) | 0.88  (0.03-7.38) | 0.013 | 0.086 | 0.545 | 0.005 |
|  |  |  |  |  |  |  |  |  |
|  |  |  |  |  |  |  |  |  |
| **Hsa-miR-5096** |  |  |  |  |  |  |  |  |
| Median (range) | 54.31  (4.98-264.79) | 22.94  (6.30-47.44) | 19.38  (1.51-323.08) | 28.76  (1.51-323.08) | 0.003 | 0.532 | 0.0312 | 0.002 |
|  |  |  |  |  |  |  |  |  |
|  |  |  |  |  |  |  |  |  |
| **Has-Let-7i-3p** |  |  |  |  |  |  |  |  |
| Median (range) | 1.42  (0.48-6.83) | 0.28  (0.10-1.14) | 0.67  (0.08-6.47) | 0.91  (0.08-6.83) | <0.001 | 0.065 | 0.046 | <0.001 |
|  |  |  |  |  |  |  |  |  |
|  |  |  |  |  |  |  |  |  |
| **P1** |  |  |  |  |  |  |  |  |
| Median (range) | 50.5  (2.65-736.43) | 7.89  (1.27-64.39) | 21.23  (0.12-2332.32) | 28.42  (0.12-2332.32) | 0.003 | 0.151 | 0.144 | 0.001 |
|  |  |  |  |  |  |  |  |  |
|  |  |  |  |  |  |  |  |  |
| **P2** |  |  |  |  |  |  |  |  |
| Median (range) | 85.16 (6.19-741.88) | 6.39 (0.67-32.05) | 15.55 (0.31-2091.76) | 35.06 (0.31-2091.76) | <0.001 | 0.086 | 0.024 | <0.001 |
|  |  |  |  |  |  |  |  |  |
|  |  |  |  |  |  |  |  |  |
| **P3** |  |  |  |  |  |  |  |  |
| Median (range) | 50.53  (2.65-736.43) | 7.89  (1.27-64.39) | 21.23  (0.12-2332.33) | 28.42  (0.12-2332.33) | 0.003 | 0.151 | 0.144 | 0.001 |
|  |  |  |  |  |  |  |  |  |
|  |  |  |  |  |  |  |  |  |
| **P** |  |  |  |  |  |  |  |  |
| Median (range) | 130.11  (2.69-3111.11) | 1.93  (0.13-25.06) | 18.63  (0.01-15100.2) | 34.28  (0.01-15100.2) | <0.001 | 0.052 | 0.097 | <0.001 |
|  |  |  |  |  |  |  |  |  |

**Table legend:** Kruskal-Wallis test was used to compare the three groups. Dunn test was used for post-hoc comparisons.

NB: ID Patient=13 in ctrl group was always not evaluable for MIRNA and was excluded.

**Supplementary table S3** Combined predictors (P1, P2, P3 and P) show significant comparable AUCs for ^18^FDG/PET positivity predictions.

| **Clinical Endpoint** | **Predictor** | **AUC (95%CI)** | **Compared predictor** | **AUC (95%CI)** | **p-value** |
| --- | --- | --- | --- | --- | --- |
| **^18^FDG/PET** **positivity** | **miR-4311** | 0.80  62 (0.66-0.94) | **miR-5096** | 0.8246 (0.69-0.95) | 0.8257 |
|  |  |  | **let-7i-3p** | 0.9477 (0.88-1.99) | 0.0541 |
|  |  |  | **P1** | 0.9231 (0.83-1.00) | 0.0068 |
|  |  |  | **P2** | 0.9508 (0.89-1.00) | 0.0285 |
|  |  |  | **P3** | 0.8462 (0.71-0.97) | 0.3794 |
|  |  |  | **P** | 0.9323 (0.85-1.00) | 0.0188 |
|  | **miR-5096** | 0.8246 (0.69-0.95) | **let-7i-3p** | 0.9477 (0.88-1.99) | 0.0913 |
|  |  |  | **P1** | 0.9231 (0.83-1.00) | 0.1731 |
|  |  |  | **P2** | 0.9508 (0.89-1.00) | 0.0310 |
|  |  |  | **P3** | 0.8462 (0.71-0.97) | 0.0642 |
|  |  |  | **P** | 0.9323 (0.85-1.00) | 0.0505 |
|  | **let-7i-3p** | 0.9477 (0.88-1.99) | **P1** | 0.9231 (0.83-1.00) | 0.534 |
|  |  |  | **P2** | 0.9508 (0.89-1.00) | 0.9260 |
|  |  |  | **P3** | 0.8462 (0.71-0.97) | 0.1313 |
|  |  |  | **P** | 0.9323 (0.85-1.00) | 0.6598 |
|  | **P1** | 0.9231 (0.83-1.00) | **P2** | 0.9508 (0.89-1.00) | 0.4841 |
|  |  |  | **P3** | 0.8462 (0.71-0.97) | 0.1240 |
|  |  |  | **P** | 0.9323 (0.85-1.00) | 0.7333 |
|  | **P2** | 0.9508 (0.89-1.00) | **P3** | 0.8462 (0.71-0.97) | 0.0217 |
|  |  |  | **P** | 0.9323 (0.85-1.00) | 0.3671 |
|  | **P3** | 0.8462 (0.71-0.97) | **P** | 0.9323 (0.85-1.00) | 0.0259 |
| **6-months PFS** | **miR-4311** | 0.7931 (0.58-1.00) | **miR-5096** | 0.8966 (0.76-1.00) | 0.4505 |
|  |  |  | **Let-7i-3p** | 0. 5402 (0.23-0.85) | 0.0205 |
|  |  |  | **P1** | 0.7126 (0.45-0.97) | 0.1413 |
|  |  |  | **P2** | 0.8276 (0.69-0.97) | 0.6751 |
|  |  |  | **P3** | 0.8062 (0.73-1.00) | 0.3881 |
|  |  |  | **P** | 0.8621 (0.74-0.98) | 0.3868 |
|  | **miR-5096** | 0.8966 (0.76-1.00) | **Let-7i-3p** | 0. 5402 (0.23-0.85) | 0.0804 |
|  |  |  | **P1** | 0.7126 (0.45-0.97) | 0.2824 |
|  |  |  | **P2** | 0.8276 (0.69-0.97) | 0.3574 |
|  |  |  | **P3** | 0.8062 (0.73-1.00) | 0.6908 |
|  |  |  | **P** | 0.8621 (0.74-0.98) | 0.6849 |
|  | **let-7i-3p** | 0. 5402 (0.23-0.85) | **P1** | 0.7126 (0.45-0.97) | 0.0134 |
|  |  |  | **P2** | 0.8276 (0.69-0.97) | 0.0628 |
|  |  |  | **P3** | 0.8062 (0.73-1.00) | 0.0554 |
|  |  |  | **P** | 0.8621 (0.74-0.98) | 0.0254 |
|  | **P1** | 0.7126 (0.45-0.97) | **P2** | 0.8276 (0.69-0.97) | 0.3180 |
|  |  |  | **P3** | 0.8062 (0.73-1.00) | 0.2249 |
|  |  |  | **P** | 0.8621 (0.74-0.98) | 0.1724 |
|  | **P2** | 0.8276 (0.69-0.97) | **P3** | 0.8062 (0.73-1.00) | 0.2425 |
|  |  |  | **P** | 0.8621 (0.74-0.98) | 0.1724 |
|  | **P3** | 0.8062 (0.73-1.00) | **P** | 0.8621 (0.74-0.98) | 0.8144 |
| **12-months OS** | **miR-4311** | 0.7143 (0.45-0.97) | **miR-5096** | 0.8929 (0.72-1.00) | 0.3553 |
|  |  |  | **Let-7i-3p** | 0.5179 (0.04-0.99) | 0.1699 |
|  |  |  | **P1** | 0.6607 (0.31-1.00) | 0.4202 |
|  |  |  | **P2** | 0.7857 (0.63-0.94) | 0.5713 |
|  |  |  | **P3** | 0.8393 (0.70-0.97) | 0.3605 |
|  |  |  | **P** | 0.8393 (0.70-0.97) | 0.2196 |
|  | **miR-5096** | 0.8929 (0.72-1.00) | **Let-7i-3p** | 0.5179 (0.04-0.99) | 0.2306 |
|  |  |  | **P1** | 0.6607 (0.31-1.00) | 0.3439 |
|  |  |  | **P2** | 0.7857 (0.63-0.94) | 0.2492 |
|  |  |  | **P3** | 0.8393 (0.70-0.97) | 0.4795 |
|  |  |  | **P** | 0.8393 (0.70-0.97) | 0.6272 |
|  | **let-7i-3p** | 0.5179 (0.04-0.99) | **P1** | 0.6607 (0.31-1.00) | 0.1025 |
|  |  |  | **P2** | 0.7857 (0.63-0.94) | 0.2722 |
|  |  |  | **P3** | 0.8393 (0.70-0.97) | 0.2175 |
|  |  |  | **P** | 0.8393 (0.70-0.97) | 0.1534 |
|  | **P1** | 0.6607 (0.31-1.00) | **P2** | 0.7857 (0.63-0.94) | 0.4768 |
|  |  |  | **P3** | 0.8393 (0.70-0.97) | 0.3510 |
|  |  |  | **P** | 0.8393 (0.70-0.97) | 0.2476 |
|  | **P2** | 0.7857 (0.63-0.94) | **P3** | 0.8393 (0.70-0.97) | 0.2149 |
|  |  |  | **P** | 0.8393 (0.70-0.97) | 1.0000 |
|  | **P3** | 0.8393 (0.70-0.97) | **P** | 0.8393 (0.70-0.97) | 1.0000 |

**Table Legend S3:** Comparison of AUC of single miRNAs and combined predictors for different outcomes (^18^FDG/PET positivity, 6-months PFS and 12-months OS). P1 (hsa-miR-4311* hsa-let-7i-3p); P2 (hsa-mir-5096*hsa-let-7i-3p); P3 (hsa-miR-4311* hsa-mir-5096); P (hsa-miR-4311*hsa-mir-5096*hsa-let-7i-3p). AUC: Area Under the Curve; Sens.(%); sensitivity percentage; Spec.(%): specificity percentage; C.I: Confidence interval.

**Supplementary Table S4: Univariable and multivariable Cox regression models of Predictors for PFS including clinical parameters**

| P1AC: >2.1 vs ≤2.1 | 1.37 /0.51-3.61) | 0.527 |  |  |
| --- | --- | --- | --- | --- |
|  |  |  |  |  |
| P2BC: >123.3 vs ≤123.3 | 2.19 (0.83-5.73) | 0.110 |  |  |
|  |  |  |  |  |
| P3AB: >142.9 vs ≤142.9 | 1.95 (1.26-10.42) | 0.017 |  |  |
|  |  |  |  |  |
| P: >108.3 vs ≤108.3 | 2.51 (0.95-6.58) | 0.061 |  |  |
|  |  |  |  |  |

**Table legend:** HR:Hazard ratio; C.I: Confidence interval; § Ki67 and grading subgroup were collinear and in the multivariable model only grading was considered in multivariable model; # Specific cut off for 3-months PFS was calculated through ROC curve; @ P3AB not included in the multivariable model because it was a combination that includes hsa-miR-5096

**Supplementary figure S3** Receiver Operating Characteristic (ROC) and Kaplan–Meier (KM) analysis of combined predictors (P1, P2, P3 and P) for 6-months progression free survival (PFS) and 12-months overall survival (OS).

**
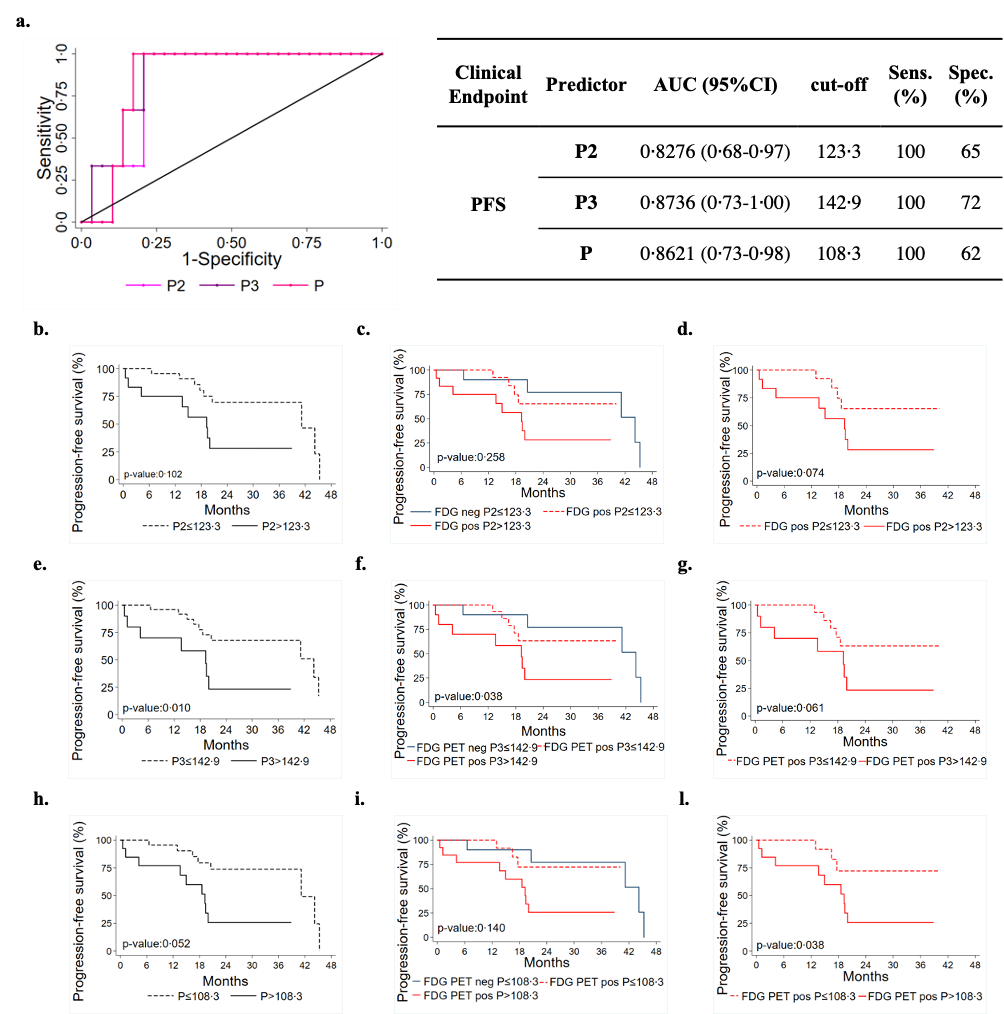
**

**Figure legend:** The performance of circulating P2, P3 and P in predicting Progression Free Survival (PFS) outcome in PAN-NEN patients treated with ^177^Lu-DOTATATE (a-l). (a) Combined ROC curve of P2, P3 and P, with significantly high AUCs. Associated table reports AUCs values, the identified cut-off and sensitivity and specificity percentages of proposed biomarkers. (b-l) Kaplan–Meier analysis (KM) for Progression Free Survival (PFS) of: (b) P2 (cut-off: 123·3); (c) P2 (cut-off: 123·3), according to ^18^F-FDG/PET-CT outcome; (d) P2 (cut-off: 123·3), in ^18^F-FDG/PET-CT positive patients. (e) P3 (cut-off: 142·9) (f) KM analysis of P3 (cut-off: 142·9), according to ^18^F-FDG/PET-CT outcome; (g) P3 (cut-off: 142·9), in ^18^F-FDG/PET-CT positive subgroup; (h) P (cut-off: 108·3); (I) P (cut-off: 108·3), according to ^18^F-FDG/PET-CT outcome; (l) P (cut-off: 108·3), in ^18^F-FDG/PET-CT positive subgroup.

**Supplementary figure S4** Time dependent AUC curve for P, P2 and P3.

**
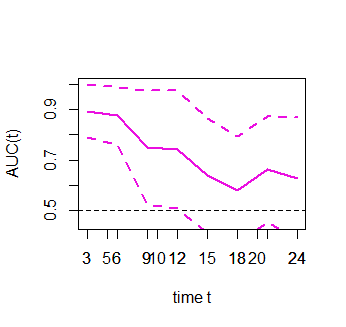

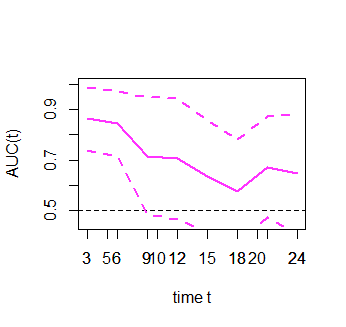

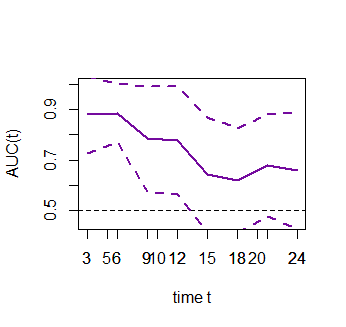
**

**Figure legend:** TimeROC package in R software was used to provide an estimation of time-dependent ROC curve and area under time dependent ROC curve (AUC) in the presence of censored data. We provide statistical analyses with the explorative intent to provide pilot evidence of its prognostic value and we fully agree with the Reviewer that validation of target and reference miRNA, as well of the proposed cut-off is required. Predictors P, P2 and P3 AUCS over time (range : 3-24 month) with 95% confidence interval (C.I).
