# Supplementary material for: Circulating hsa-miR-5096 predicts 18F-FDG PET/CT positivity and modulates somatostatin receptor 2 expression: a novel miR-based assay for pancreatic neuroendocrine tumors
Source: Front Oncol. 2023 May 23;13:1136331. doi: 10.3389/fonc.2023.1136331 (PMC10242108; doi:10.3389/fonc.2023.1136331)
Supplement: Supplementary file 2 [file DataSheet_2.pdf]

AND Tool – user manual

http://sourceforge.net/p/andtool

ANALYSIS NUCLEI DAB

-AND-

TOOL

v1.0 – January 2021

Filippo Piccinini, PhD

IRCCS Istituto Romagnolo per lo Studio dei Tumori (IRST) "Dino Amadori"
Cancer Research Hospital
IRST IRCCS, Meldola (FC), Italy
filippo.piccinini85@gmail.com
www.filippopiccinini.it

01. BRIEF DESCRIPTION

Analysis Nuclei DAB Tool (hereafter just named as “AND-Tool”) is a Graphical User Interface (GUI) designed for automatically analysing microscopy 2D images representing cells with nuclei stained using a staining of interest, typically a DAB dye. The images considered are those acquired with a widefield microscope with a 20x objective and an embedded Red-Green-Blue (RGB) camera. The tool first segment the nuclei using the FastBlue channel and the nuclear-signal-of-interest (nsoi, e.g. DAB) channel, and then computes statistics (e.g. single nuclei intensity and frequency of nuclei) by subdividing the image into three regions of interest (ROIs) according to the FastRed channel: dark-red ROIs, light-pink ROIs and white ROIs. Two types of nuclei are considered: the ones positive for the nuclear-signal-of-interest staining, and the ones considered as nuclei because visible in the FastBlue channel but not positive for the nuclear-signal-of-interest staining.

AND-Tool is written in MATLAB (The MathWorks, Inc., Massachusetts, USA) and the source code and standalone versions are available for download at: http://sourceforge.net/p/andtool.

02. LICENSE

The software and all the materials available at:
http://sourceforge.net/p/andtool are copyright protected.

Copyright (©) 2020, Filippo Piccinini. All rights reserved.
AND-Tool is licensed under the:
3-clause BSD License

The exact license text is:

Redistribution and use in source and binary forms, with or without modification, are permitted provided that the following conditions are met:

- Redistributions of source code must retain the above copyright notice, this list of conditions and the following disclaimer.
- Redistributions in binary form must reproduce the above copyright notice, this list of conditions and the following disclaimer in the

documentation and/or other materials provided with the distribution.

- Neither the name of the Institutes supporting this project nor the names of its contributors may be used to endorse or promote products derived from this software without specific prior written permission.

THIS SOFTWARE IS PROVIDED BY THE COPYRIGHT HOLDERS AND CONTRIBUTORS "AS IS" AND ANY EXPRESS OR IMPLIED WARRANTIES, INCLUDING, BUT NOT LIMITED TO, THE IMPLIED WARRANTIES OF MERCHANTABILITY AND FITNESS FOR A PARTICULAR PURPOSE ARE DISCLAIMED. IN NO EVENT SHALL <COPYRIGHT HOLDER> BE LIABLE FOR ANY DIRECT, INDIRECT, INCIDENTAL, SPECIAL, EXEMPLARY, OR CONSEQUENTIAL DAMAGES (INCLUDING, BUT NOT LIMITED TO, PROCUREMENT OF SUBSTITUTE GOODS OR SERVICES; LOSS OF USE, DATA, OR PROFITS; OR BUSINESS INTERRUPTION) HOWEVER CAUSED AND ON ANY THEORY OF LIABILITY, WHETHER IN CONTRACT, STRICT LIABILITY, OR TORT (INCLUDING NEGLIGENCE OR OTHERWISE) ARISING IN ANY WAY OUT OF THE USE OF THIS SOFTWARE, EVEN IF ADVISED OF THE POSSIBILITY OF SUCH DAMAGE.

03. SYSTEM REQUIREMENTS

- *Source code*: AND-Tool source code is written in MATLAB (The MathWorks, Inc., Natick, MA, USA) and works on Windows, Macintosh and Linux operating systems. It requires a MATLAB R2020a. the Image Processing Toolbox 11.1, the Statistics and Machine Learning Toolbox 11.7, or later versions.
- *Win-64 Standalone Version*: the standalone version of the tool does not require a MATLAB licence, but it currently (September 2020) works just on Windows 64-bit systems. Windows 10 (or later versions) with a CPU i3, RAM 4 GB, or higher performance computers are suggested.

04. INSTALLATION

There are two ways to use the AND-Tool:

- *Source code*: For who has a MATLAB licence, we provide the source code. In this case, it is enough to download the source code and extract the files from "ANDTool_v#.zip" to a custom folder. Then,

start MATLAB and select the folder containing the AND-Tool files. Upon typing “START_GUI” in the MATLAB Command Window the AND-Tool GUI will pop-up.

- *Win-64 Standalone Version*: To install and use the software from the standalone version you need the Administrator rights of the computer.
  - *(a)* unzip the file: "AND_v_*_*_WIN64bit_Standalone.zip".
  - *(b)* You need an Internet connection active.
  - *(c)* Double-click on the file: "MyAppInstaller_web.exe" and follow the steps.
  - *(d)* Once the software is installed, to run the software: Double-click on the "AND_v_*_*_.exe" file, typically automatically generated in the "application" folder created in the selected path of the installation folder.

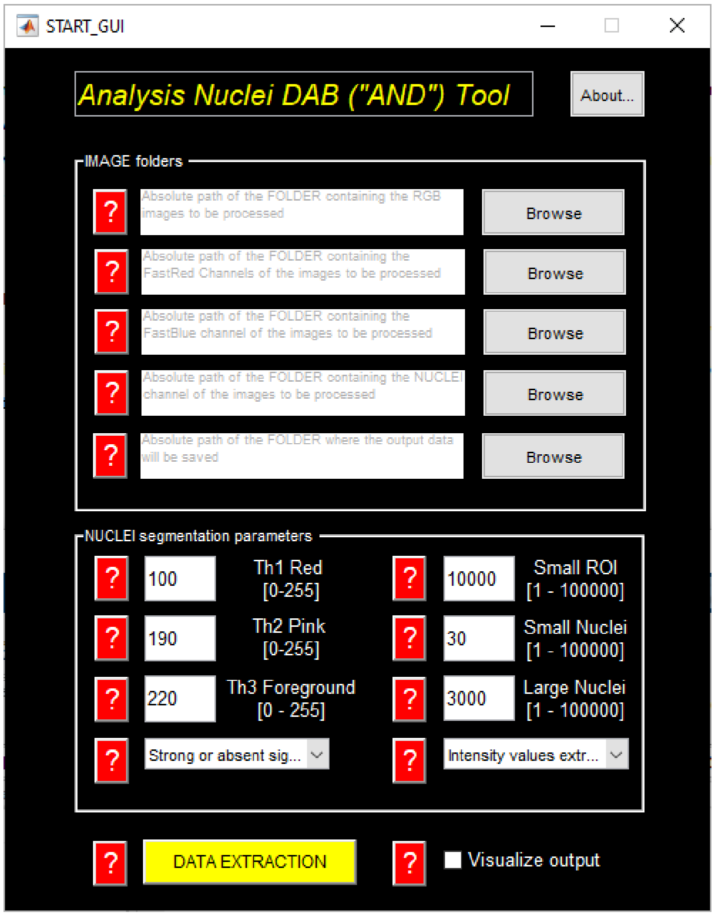

Fig. 1: The AND-Tool GUI.

05. USAGE

The AND-Tool's GUI is designed for automatically analysing microscopy 2D images representing cells with nuclei stained using a nuclear-signal-of-interest, typically a DAB dye. The images considered are those acquired with a widefield microscope with a 20x objective and an embedded RGB camera, but adjusting the parameters is possible to analyse also images acquired using different objective lens..

The tool requires as input the:

- original RGB images (i.e. RGB-type images)

plus, the:

- FastRed channel (i.e. mono-channel gray-level images)
- FastBlue channel (i.e. mono-channel gray-level images)
- nuclear-signal-of-interest (i.e. DAB) channel (mono-channel gray-level images)

of each image to be analysed. Precisely, the tool requires to set in the first four fields of the GUI the path of the "RGB", "FastRed", "FastBlue" and "DAB" folders, containing the images to be analysed.

Note, the folders must contain images only, in a standard format like ".tiff" or ".png". All the images in the "RGB" image folder will be analysed. Accordingly, if your interest is to analyse a single specific image, include just that one in the folder set as "RGB" image folder.

06. IMAGE PRE-PROCESSING

- Vignetting correction: To improve the results is better to pre-process the original RGB images correcting for vignetting. One way is to use Fiji and run:

  "ImageJ/Fiji" -> "Process" -> "Subtract Background…"

  Note: set the "Rolling Ball Radius [pixels]" approximatively to the radius of the objects of interest.

- From "RGB" to "FastRed FastBlue DAB": as we wrote, the AND-Tool is designed for analysing RGB images acquired using a widefield microscope embedded with an RGB camera. To convert an RGB image into the 3 channels FastRed, FastBlue and DAB, you can use the Fiji function:

  "ImageJ/Fiji" -> "Image" -> "Colour Deconvolution" -> "FastRed FastBlue DAB"

07. GETTING STARTED

AND-Tool is designed for automatically analysing microscopy 2D images representing cells with nuclei stained using a nuclear-signal-of-interest (e.g. DAB) dye.

The tool requires the as input the original RGB images plus, the FastRed channel, FastBlue channel, DAB channel of each image to be analysed. Accordingly, first of all, all the original RGB images are converted into the triple FastRed, FastBlue, and DAB channels using the “Colour Deconvolution" Fiji function.

All the images are then loaded and converted from the tool into 8-bits (i.e. intensity values from 0 to 255). The tool then subdivides each image in ROIs defined using the FastRed channel and 2 thresholds defined by the user (hereafter named Th1 and Th1):

- the “Dark-Red” ROIs, with intensity values of the FastRed channel between 0 and Th1
- the “Light-Pink” ROIs, with intensity values of the FastRed channel between Th1 and Th2
- the “White” ROIs, with intensity values of the FastRed channel between Th2 and 255.

Note that the ROIs are then smoothed with some morphological operations.

The nuclei are then segmented using the FastBlue channel. After that, the nuclear-signal-of-interest channel (typically the DAB images) is used to detect the nuclei positive to the dye of interest. Note that nuclei positive for the nuclear-signal-of-interest, but not for the FastBlue channel, will be considered as nuclei even if not present in the list of nuclei detected using the FastBlue channel. Furthermore, the images typically considered are those acquired with a widefield microscope with a 20x objective and an embedded RGB camera with a good quality nuclear-signal-of-interest channel, but adjusting the parameters is possible to analyse also images acquired using different objective lens and images with absent, faint and strong intensity of the nuclear-signal-of-interest channel (to understand better how to use the AND-Tool see the example in the following sections).

Finally, AND-Tool computes statistics, for instance the single nuclei intensity and frequency of nuclei in the different ROIs and saves as output a table reporting for each nucleus the mean intensity value.

08. GUI’S PARAMETERS

What follows is the list of parameters that can be set using the AND-Tool’s GUI:

- “*RGB images - path*”: to define the absolute path of the folder containing the RGB images (i.e. RGB-type images) to be processed.
- “*FastRed images - path*”: to define the absolute path of the folder containing the FastRed images (i.e. mono-channel gray-level images typically obtained using the Colour Deconvolution plugin of Fiji) to be processed.
- “*FastBlue images - path*”: to define the absolute path of the folder containing the FastBlue images (i.e. mono-channel gray-level images typically obtained using the Colour Deconvolution plugin of Fiji) to be processed.
- “*CHANNEL-OF-INTEREST images - path*”: to define the absolute path of the folder containing the nuclear-signal-of-interest images (typically, the DAB channel in mono-channel gray-level image format typically obtained using the Colour Deconvolution plugin of Fiji) to be processed.
- “*Output Folder - path*”: to define the absolute path of the folder then used from the tool to save the output tables and images.
- “*Th1 Red*”: Threshold value (Th1) value defining the highest level for the "Dark-Red" ROIs when analysing the FastRed images. The value must be a positive value between 1 and 255.
- “*Th2 Pink*”: Threshold value (Th2) value defining the highest level for the "Light-Pink" ROIs when analysing the FastRed images. The value must be a positive value between 1 and 255 and must be higher than Th1.
- “*Th3 Foreground Intensity*”: Threshold value (Th3) defining the maximum intensity value to be considered when defining the nuclei in the nuclear-signal-of-interest images. All pixels with original intensity value higher than this threshold are excluded. The value must be a positive value between 1 and 255.
- “*Small ROI*”: Threshold value defining the smaller number of pixels considered when defining the red, pink and white ROIs. The value must be a positive value between 1 and 100000 pixels.
- “*Small Nuclei*”: Threshold value defining the smaller number of pixels considered when detecting the nuclei. The value must be a positive value between 1 and 100000 pixels.
- “*Large Nuclei*”: Threshold value defining the maximum number of pixels considered when detecting the nuclei. The value must be a positive value between 1 and 100000.
- “*Channel selected for intensity statistics*”: Image from which to extract the intensity value considered for each segmented nucleus. Currently, two options are available:

  (*a*) “Intensity values extracted from the original RGB images gray converted”;

  (*b*) “Intensity values extracted from the single FastRed-FastBlue-Other channels”.

If the first option is selected, a gray conversion of the original RGB images will be used to extract the intensity value. If the second option is selected, the FastBlue images are used for extracting the intensity value of the blue nuclei, and the nuclear-signal-of-interest images are used to extract the intensity value for the other nuclei.

- “*Visualize output*”: If enabled, the temporary images obtained during computation are visualised during the processing of the data.
- “*Nuclei channel intensity level*”: To adjust the threshold between background and foreground of the nuclear-signal-of-interest channel (i.e. DAB images):
  (*a*) "Faint (but present) signal" enables a series of filters to increase the threshold between background and foreground and consider more bright spots as foreground;
  (*b*) "Strong or absent signal" (default) considers as foreground just the darker spots of the nuclear-signal-of-interest images.

09. NOTE OF USAGE

What follows is the general scheme of usage of the AND-Tool:

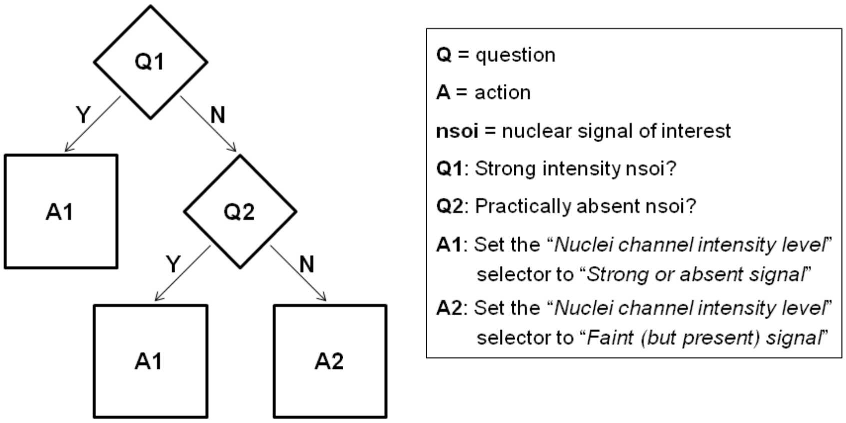

Fig. 1: The AND-Tool GUI.

For instance, in case of a strong nuclear-signal-of-interest:

RGB

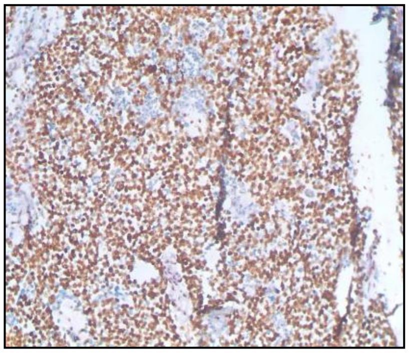

DAB

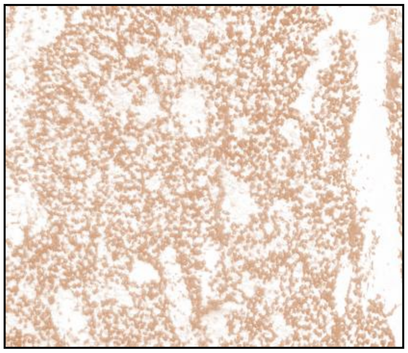

or an absent nuclear-signal-of-interest:

RGB

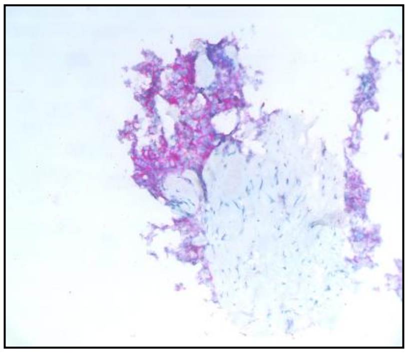

DAB

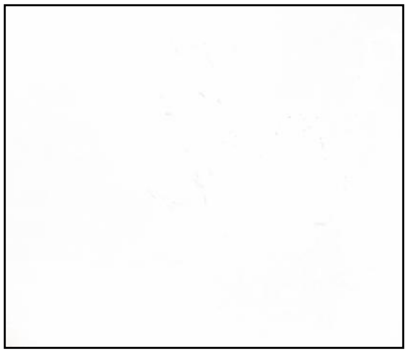

run the AND-Tool simply by setting the “Nuclei channel intensity level” to "Strong or absent signal".

In case of a faint nuclear-signal-of-interest:

RGB

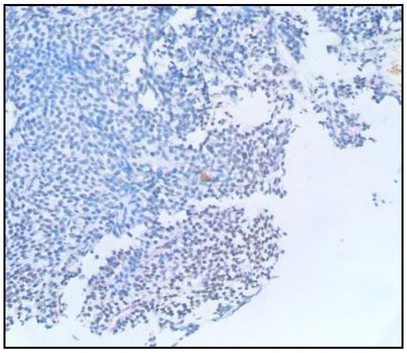

DAB

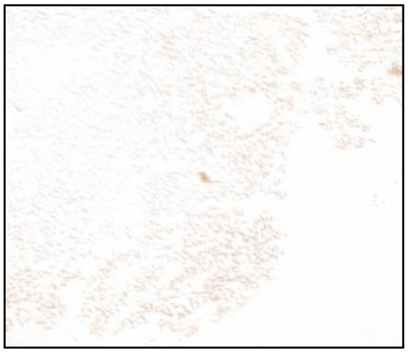

run the AND-Tool by setting the “Nuclei channel intensity level” to "Faint (but present) signal" and carefully set Th3 to a level appropriate to exclude from the foreground the pixels with an high intensity value (otherwise wrongly considered as a correct nuclear signal).

In case of a RGB images presenting positive nuclei, showing a strong dark intensity for the nuclear-signal-of-interest, but a practically absent signal in the specific nuclear-signal-of-interest images probably for problem occurred when using the Colour Deconvolution plug-in, a possible solution is to set for the “*CHANNEL-OF-INTEREST images - path*” parameter the folder of the original “*RGB images*” (that internally will be automatically converted in grey-levels when used for evaluating the nuclear-signal-of-interest) and then carefully set Th3 to a level appropriate to exclude from the foreground the pixels with an high intensity value (otherwise wrongly considered as a correct nuclear signal).

RGB

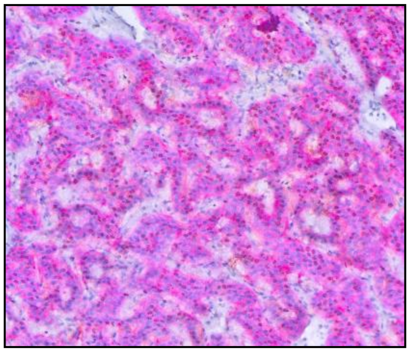

DAB

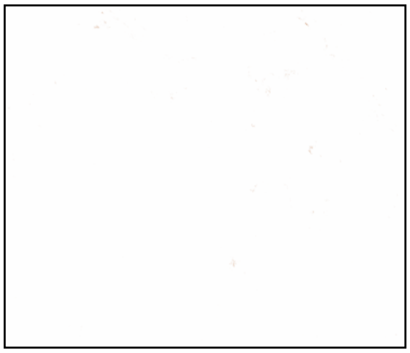

RGB (gray-converted and thresholded)

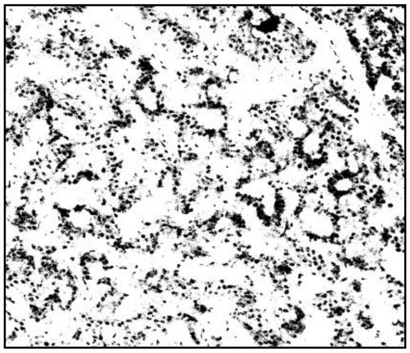

<u>10. CONTACTS</u>

By reading this manual you got a more complete overview of the various analysis options offered by the AND-Tool. Please, also visit the AND-Tool website: http://sourceforge.net/p/andtool, and If you need

further information or you have special requests, remember that we are open to collaborations! In that case contact Filippo Piccinini at: filippo.piccinini85@gmail.com
